# Reaction Pathway for Coke-Free Methane Steam Reforming on a Ni/CeO_2_ Catalyst: Active Sites and the Role of Metal–Support Interactions

**DOI:** 10.1021/acscatal.1c01604

**Published:** 2021-06-23

**Authors:** Agustín Salcedo, Pablo G. Lustemberg, Ning Rui, Robert M. Palomino, Zongyuan Liu, Slavomir Nemsak, Sanjaya D. Senanayake, José A. Rodriguez, M. Verónica Ganduglia-Pirovano, Beatriz Irigoyen

**Affiliations:** † Departamento de Ingeniería Química, Facultad de Ingeniería, Universidad de Buenos Aires (UBA), Ciudad Universitaria, C1428EGA Buenos Aires, Argentina; ‡ Instituto de Tecnologías del Hidrógeno y Energías Sostenibles (ITHES, CONICET-UBA), Ciudad Universitaria, C1428EGA Buenos Aires, Argentina; § Instituto de Catálisis y Petroleoquímica (ICP, CSIC), 28049 Madrid, Spain; ∥ Instituto de Física Rosario (IFIR, CONICET-UNR), S2000EKF Rosario, Santa Fe, Argentina; ⊥ Chemistry Division, Brookhaven National Laboratory, Upton, New York 11973, United States; # Advanced Light Source, Lawrence Berkeley National Laboratory, Berkeley, California 94720, United States

**Keywords:** methane, steam reforming, hydrogen, nickel, ceria, DFT

## Abstract

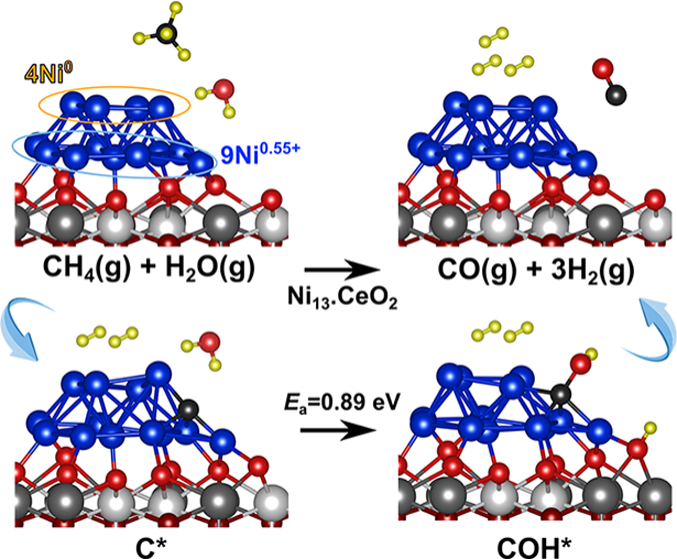

Methane steam reforming (MSR) plays a key role in the production of syngas and hydrogen from natural gas. The increasing interest in the use of hydrogen for fuel cell applications demands development of catalysts with high activity at reduced operating temperatures. Ni-based catalysts are promising systems because of their high activity and low cost, but coke formation generally poses a severe problem. Studies of ambient-pressure X-ray photoelectron spectroscopy (AP-XPS) indicate that CH_4_/H_2_O gas mixtures react with Ni/CeO_2_(111) surfaces to form OH, CH_*x*_, and CH_*x*_O at 300 K. All of these species are easy to form and desorb at temperatures below 700 K when the rate of the MSR process is accelerated. Density functional theory (DFT) modeling of the reaction over ceria-supported small Ni nanoparticles predicts relatively low activation barriers between 0.3 and 0.7 eV for complete dehydrogenation of methane to carbon and the barrierless activation of water at interfacial Ni sites. Hydroxyls resulting from water activation allow for CO formation via a COH intermediate with a barrier of about 0.9 eV, which is much lower than that through a pathway involving lattice oxygen from ceria. Neither methane nor water activation is a rate-determining step, and the OH-assisted CO formation through the COH intermediate constitutes a low-barrier pathway that prevents carbon accumulation. The interactions between Ni and the ceria support and the low metal loading are crucial for the reaction to proceed in a coke-free and efficient way. These results pave the way for further advances in the design of stable and highly active Ni-based catalysts for hydrogen production.

## 1 Introduction

Methane steam reforming (MSR, CH_4_ + H_2_O ⇄ 3H_2_ + CO) is the main route for the large-scale industrial manufacture of hydrogen, primarily used for the synthesis of ammonia and methanol, among other commodities,^1^ as well as the hydrocracking of long-chain hydrocarbons in petroleum refineries.^2^ In a typical industrial reformer, the MSR reaction is carried out at 800–1000 °C and 14–20 atm, with a H_2_O/CH_4_ ratio of ∼2.5.^1,3^ Environmental concerns about air pollution and greenhouse gases have renewed the interest in using hydrogen as a clean energy carrier for automotive applications through its electrochemical conversion in fuel cell systems, which produces water as the only byproduct. However, the severe reaction conditions of industrial MSR result in elevated capital and operating costs, which are prohibitive for small-scale fuel cell applications. Several alternative reactions have been proposed, such as methane dry reforming and partial oxidation, but their lower H_2_/CO ratio compared to that of MSR makes them unfit for fuel cell applications that require high-purity H_2_.^4−6^ Therefore, it is necessary to improve MSR technology to reduce heating and steam requirements and achieve cost-efficient H_2_ manufacture. In this sense, the capability to operate fuel cells at ambient pressure^7^ and the development of hydrogen-selective membrane reactors^8−10^ represent an opportunity to increase the thermodynamically limited conversion imposed by the endothermicity of the MSR reaction,^11^ allowing for both lower operating temperatures (500–600 °C) and lower steam-to-methane ratios while maintaining good H_2_ yield. Commercial catalysts in industrial reforming units, typically consisting of nickel on magnesium or aluminum oxide supports, are designed to withstand high-temperature operations without losing strength and thus prioritize stability and thermal resistance over surface area.^1^ In addition, they are prone to deactivation by coking, sintering, and sulfur poisoning.^12^ Noble metals such as Pt, Rh, and Pd are also active for MSR but more expensive than Ni.^11^ Therefore, the present challenge is to develop novel Ni-based catalysts to carry out the MSR reaction with high conversion at mild operating conditions for fuel cell applications. Among recently proposed alternatives, low-loaded Ni-impregnated CeO_2_ catalysts have shown potential as promising candidates, showing improved coking and sintering resistance and excellent performance in experiments carried out at 600 °C and ambient pressure.^13,14^

A complete understanding of the MSR reaction mechanism over ceria-supported Ni catalysts, which includes the identification of the active sites and the determination of the relevant reaction pathways, remains elusive, but it is essential to be able to modify the catalyst to enhance activity and selectivity.

Insights from density functional theory (DFT) calculations on model Ni-based MSR catalysts have so far been limited to extended Ni surfaces. It has been postulated that the activation of CH_4_ determines the overall reaction rate^15,16^ because its C–H bonds are very stable (440 kJ/mol),^17,18^ and pure metal surfaces tend to show low reactivity toward methane.^19^ However, experimental and computational studies have shown that the reactions of carbonaceous species with oxygen to form the C–O bond also involve high energy barriers and could therefore be rate-controlling.^20−26^ Furthermore, in previous combined computational and in situ spectroscopic studies, it was shown that well-dispersed small Ni nanoparticles supported on a nonreduced CeO_2_ surface can in fact activate CH_4_ at room temperature, with calculated energy barriers up to 80% lower than those for extended nickel surfaces.^27−31^ This highlights the need to consider both the effect of the nature of the support and the metal loading to fully understand the mechanism governing the MSR reaction over supported metal catalysts, which is necessary for the development of improved catalytic systems.

In general, dispersed metal nanoparticles on oxide surfaces tend to be more reactive than the individual components, showing great potential as novel catalytic materials.^32^ In low-loaded CeO_2_-supported Ni catalysts, nickel is stabilized as small particles in which the Ni atoms in direct contact with ceria are partially oxidized as a consequence of strong metal–support interactions,^27,33,34^ resulting in important changes in the chemical and catalytic properties of these systems, particularly to perform C–H and O–H bond cleavage.^27,28,30,35,36^ Furthermore, the easier reducibility of the ceria support allows it to act as an oxygen reservoir,^37^ providing unique reaction pathways such as the reverse spillover of oxygen from ceria to metal sites, which has been experimentally observed for a variety of ceria-supported metal catalysts, including Ni/CeO_2_.^38−43^ Therefore, the observed superior decoking activity of ceria-supported Ni catalysts for MSR could be ascribed to a mechanism involving the oxygen supply from the support promoting carbon removal as CO,^44,45^ in which the role of water as one of the reactants would be the refilling of the oxygen vacancies generated in the reverse spillover step. However, water-mediated carbon removal has also been discussed in the context of steam reforming of CH_4_.^46^ Whether carbon removal is assisted by oxygen from the support, from H_2_O, or from both is an essential question in the understanding of the MSR reaction mechanism.

Furthermore, since both reactants in the MSR reaction over Ni/CeO_2_ catalysts adsorb at Ni sites and are generally in H_2_O/CH_4_ ratios higher than 1, their potential competition for Ni sites should also be addressed. In this regard, CH_4_ conversion in the steam reforming reaction over Ni-impregnated Zr-doped CeO_2_ catalysts was found to continuously increase with H_2_O content, suggesting that whatever the competition, it was not detrimental to the reaction.^14^

It has been previously found that small coverages of nickel on CeO_2_(111) produce surfaces that are able to catalyze the MSR process at temperatures above 500 K with high activity and low propensity to deactivation by coke deposition.^29^ This is a remarkable catalytic performance. Here, using a combination of ambient-pressure X-ray photoelectron spectroscopy (AP-XPS) and molecular modeling based on density functional theory (DFT), we present a comprehensive study of the MSR reaction on the surface of model Ni/CeO_2_(111) catalysts and compare with results reported for the extended Ni(111) surface in the literature.^21−24,47−54^ We show that low-loaded Ni/CeO_2_ catalysts have sites with unique properties that result from the nature of both the metallic phase and the support and their interactions, which enable the facile activation of C–H and O–H bonds from CH_4_ and H_2_O, respectively. The calculated elementary dehydrogenation and oxidation steps along the MSR reaction reveal that the crucial step is the formation of a COH intermediate via the reaction of carbon atoms with OH groups, suppressing carbon deposition. This pathway presents much lower barriers than the one involving C oxidation with lattice oxygen from the ceria support and is promoted by the easy formation of OH groups through the barrierless dissociative adsorption of water at the Ni–CeO_2_ interface. The results provide molecular insight into the interplay between C and OH species in the steam reforming of methane on low-loaded Ni/CeO_2_ catalysts for which metal–support interactions are crucial to bind and activate methane and water.

## 2 Methods

### 2.1 Experiments of Ambient-Pressure XPS

The ambient-pressure XPS studies examining the interaction of CH_4_/H_2_O gas mixtures with the Ni/CeO_2_(111) surfaces were performed using instruments located at the Chemistry Division in Brookhaven National Laboratory (BNL) and at the Advanced Light Source (ALS) in Berkeley.^27−30^ In both instruments, the Ni/CeO_2_(111) surfaces were prepared and characterized following standard procedures.^27−29^ Ce metal was first evaporated onto a Ru(0001) substrate at 700 K under a background pressure of 5 × 10^–7^ Torr of O_2_, and then the sample was annealed at 800 K for a period of 10 min at the same O_2_ pressure. The CeO_2_(111) films were estimated to be ca. 4 nm thick (≈10 layers of O–Ce–O) based on the attenuation of the Ru 3d XPS signal. Ni was vapor-deposited on the as-prepared ceria films, and the admetal coverage was estimated by the attenuation of the Ce 3d XPS signal.^27−29^ The Ni/CeO_2_(111) surfaces were exposed to CH_4_, H_2_O, and CH_4_/H_2_O mixtures at temperatures between 300 and 700 K.

The AP-XPS instrument at BNL was a SPECS AP-XPS chamber equipped with a PHOIBOS 150 EP MCD-9 analyzer. Mg Kα radiation was used to collect the Ni 2p and Ce 3d spectra of the Ni/CeO_2_(111) samples under exposure to the reacting gases. The binding energies in these AP-XPS spectra were calibrated using as a reference the strongest Ce^4+^ 3d feature located at 916.9 eV.

At the ALS, the AP-XPS experiments were performed in beamline 9.3.2, which was equipped with a VG Scienta R4000 HiPP analyzer. On exposure of Ni/CeO_2_(111) to the reacting gases, the O 1s region was probed using a photon energy of 650 eV, and the C 1s, Ni 3p, and Ce 4d regions with a photon energy of 490 eV. The energy resolution in the synchrotron experiments was ∼0.2 eV. The Ce 4d photoemission lines were used for binding energy calibration based on the 122.8 eV satellite features. No evidence was found for the existence of beam damage in these AP-XPS studies.

### 2.2 Models and Computational Details

A Ni_13_ cluster adsorbed on the CeO_2_(111) surface with a (3 × 3) periodicity^34^ was used as a representative model of low-loaded ceria-supported nickel catalysts, hereafter referred to as Ni_13_·CeO_2_ (Figure S1). The size of the Ni_13_ cluster is comparable to that of Ni nanoparticles of model Ni/CeO_2_ catalysts in experimental studies^28,29^ and has metallic Ni^0^ and oxidized Ni^0.55+^ sites, both reported to be present at steam reforming conditions.^13^ The (3 × 3) CeO_2_(111) surface was modeled using a supercell with the calculated ceria bulk equilibrium lattice parameter of *a*_0_ = 5.485 Å, with six atomic layers (two O–Ce–O trilayers, TLs) separated by at least a 12 Å thick vacuum layer.

Calculations were performed within the spin-polarized density functional theory (DFT) framework as implemented in the Vienna Ab initio Simulation Package (VASP).^55,56^ The Kohn–Sham equations were solved within the generalized gradient approximation (GGA), with the Perdew–Burke–Ernzerhof (PBE) exchange–correlation functional.^57^ We treated explicitly the Ce(5s^2^5p^6^6s^2^5d^1^4f^1^), Ni(3p^6^4s^2^3d^8^), O(2s^2^2p^4^), and C(2s^2^2p^2^) valence electrons using a plane-wave basis with a cutoff energy of 415 eV, whereas the core electrons were represented with the projector-augmented wave (PAW) method.^58,59^ Total energies were calculated with a precision of 10^–6^ eV. Strong correlation effects due to charge localization were considered with the DFT + *U* approach within Dudarev’s scheme^60^ to compensate for the self-interaction error.^61−65^ The *U*_eff_ parameter was set to 4.5 eV for the Ce(4f) states.^66,67^ Long-range dispersion corrections were considered within the DFT-D3 approach.^68,69^ The oxidation state of a given Ce ion (Ce^4+^ or Ce^3+^) was determined by considering its local magnetic moment, which can be estimated by integrating the site- and angular momentum projected spin-resolved density of states over spheres with radii chosen as the Wigner–Seitz radii of the PAW potentials. The magnetic moments of the Ce^4+^ (4f^0^) and Ce^3+^ (4f^1^) ions are 0 and ∼1 μB, respectively. As for the oxidation state of the Ni atoms in the supported clusters, using Bader’s atom-in-molecule approach,^70,71^ we observed that only those Ni atoms bound to surface oxygen from the ceria support are partially oxidized. The average oxidation state of these Ni atoms was calculated as the total number of electrons transferred to the ceria support divided by the number of atoms in direct contact with the support. Full relaxation of atomic coordinates was allowed for both the Ni atoms and the Ce and O ions located in the uppermost TL, and forces were converged to 0.02 eV/Å. The ions in the bottom TL were kept fixed in their bulk positions. The Brillouin zone was sampled with a (2 × 2 × 1) *k*-point mesh using the Monkhorst–Pack scheme.^72^

Transition state (TS) structures were located using the climbing image nudged elastic band (CI-NEB) method^73^ with forces converged to 0.05 eV/Å. Harmonic frequencies were calculated for all TS structures using a finite-difference method, with displacements of ±0.015 Å in the coordinates of the adsorbates and the Ni atoms, to verify the existence of a single imaginary frequency.

## 3 Results

### 3.1 Surface Chemistry of the MSR Process on Ni/CeO_2_(111): An AP-XPS Study

Previous results of AP-XPS indicate that methane dissociates on Ni/CeO_2_(111) surfaces at room temperature (300 K) to yield surface CH_*x*_^27,28^ and that part of the adsorbed CH_4_ undergoes full decomposition that produces C atoms that react with O centers of the support to generate CO_*x*_ groups (CO_2_ or CO_3_ species). Maximum reactivity was observed on systems that had Ni coverage below 0.2 monolayer (ML). These systems were able to catalyze the MSR process at temperatures above 500 K with high activity and low propensity to deactivation by coke deposition.^29^Figure S2 shows Ni 2p and Ce 3d XPS spectra collected while exposing a Ni/CeO_2_(111) surface to 20 mTorr of methane at 300 and 700 K. At room temperature, the reaction of methane with the surface does not change the oxidation state of Ni or Ce in the system, but CH_*x*_ and CO_*x*_ groups are deposited on the catalyst.^27,28^ The reaction is observed only when Ni is added to ceria, but the total coverage of the CH_*x*_ and CO_*x*_ groups is larger than that of nickel, suggesting that methane dissociates on Ni or the Ni–ceria interface and then a part of the C-containing species migrate to the ceria.^27,28^ These adsorbed species are not stable at temperatures above 500 K, but the reaction with methane is very fast, and at an elevated temperature of 700 K, the decomposition products of methane reduce Ni^2+^ to Ni^0^ and a part of Ce^4+^ to Ce^3+^, which is accompanied by the formation of lattice oxygen vacancies. Therefore, during methane steam reforming over Ni/CeO_2_ at *T* ≥ 700 K, the dissociation of water on the O vacancies closes the catalytic cycle.^16,17^ However, as discussed below, the Ni^2+^ → Ni^0^ and Ce^4+^ → Ce^3+^ reductions were not observed under a mixture of methane and water.

**Figure 1:**
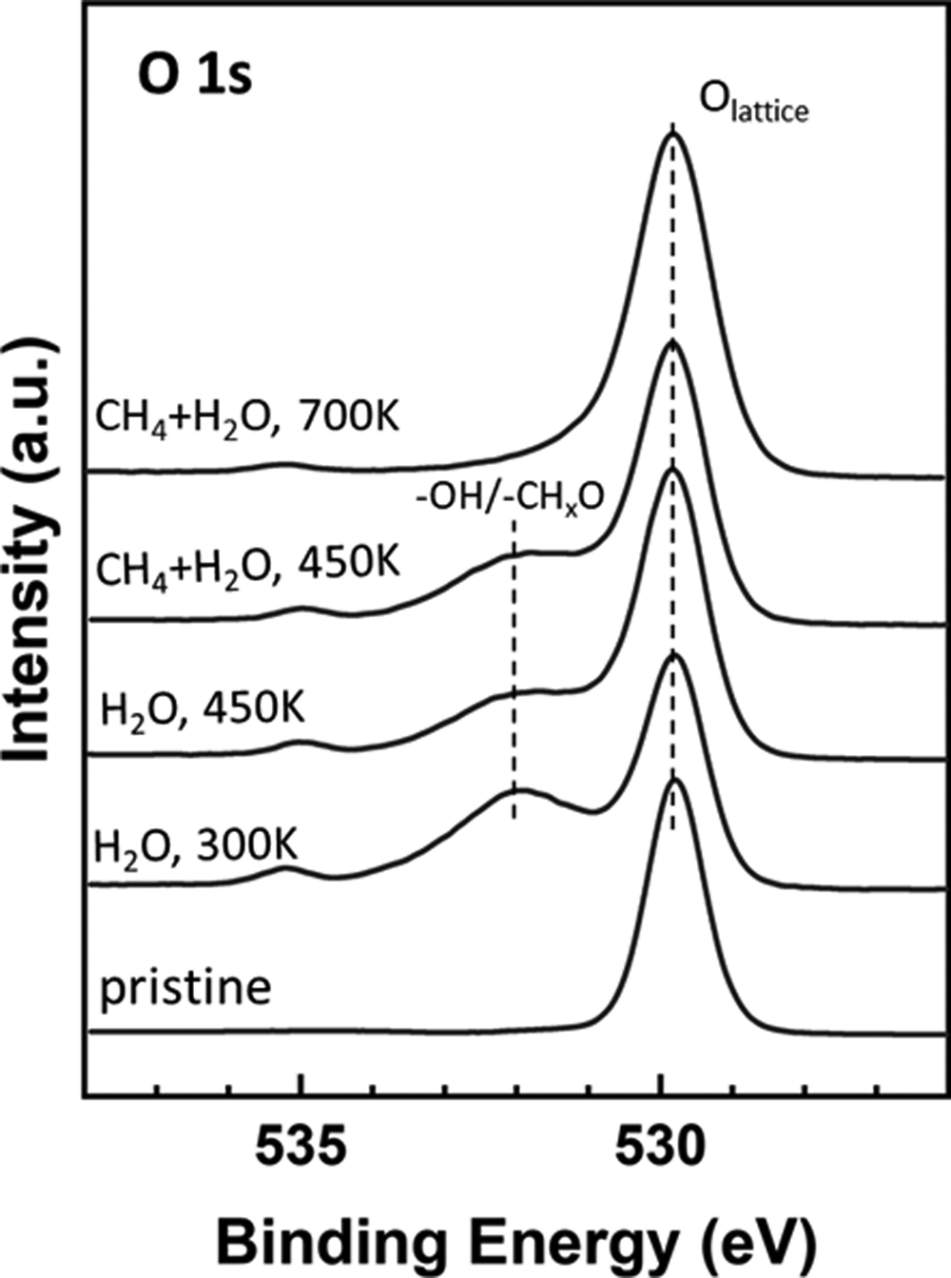
O 1s XPS spectra collected while exposing a Ni/CeO_2_(111) surface (θ_Ni_ ∼ 0.15 ML) to 100 mTorr of H_2_O at 300 and 450 K and then to a gas mixture of 25 mTorr of CH_4_ and 100 mTorr of H_2_O at 450 and 700 K.

The bottom traces in Figure 1 show O 1s XPS spectra collected while exposing a Ni/CeO_2_(111) surface to 100 mTorr of H_2_O at different temperatures. The peak at around 535 eV results from H_2_O gas. Features at around 531.8 eV denote the dissociation of the adsorbate and the deposition of OH groups on the surface.^36^ At 300 K, the total coverage of OH on the surface was in the range of 0.4–0.6 ML. The OH groups were bound to Ni and Ce cations on the substrate. The formation of Ni–OH bonds leads to a binding energy shift in the position of the Ni 2p core levels, whereas the Ce 3d core levels are not significantly affected by the dissociation of the water molecules (Figure S3). In Figure 1, there is an attenuation of the signal for surface OH groups when the temperature is increased from 300 to 450 K. Thus, the OH groups are easily formed and they do not bind strongly to the metal/oxide substrate, which are good characteristics for intermediates in a catalytic process.

**Figure 2:**
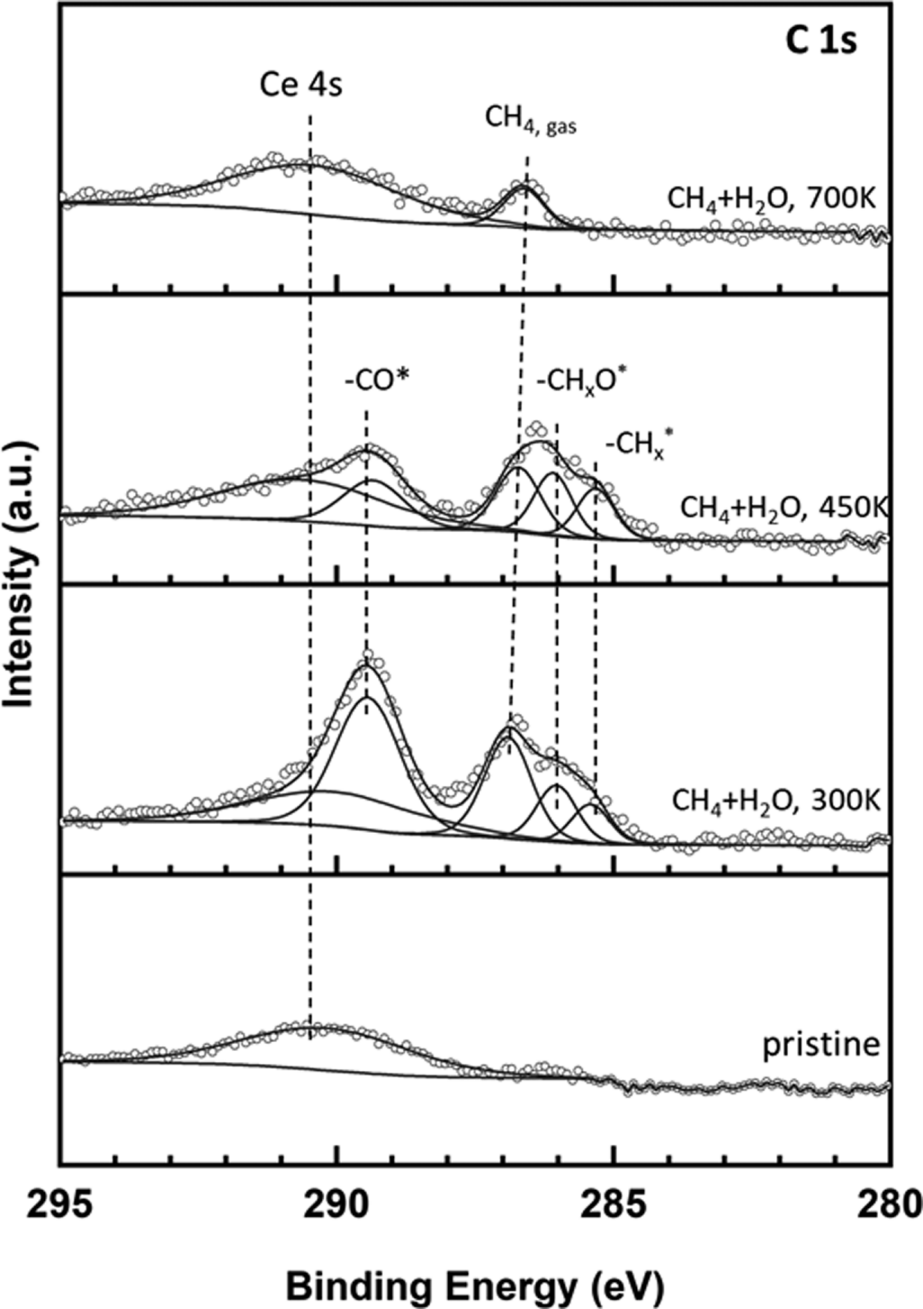
C 1s XPS spectra collected while exposing a Ni/CeO_2_(111) surface (θ_Ni_ ∼ 0.15 ML) to 25 mTorr of CH_4_ and 100 mTorr of H_2_O at the indicated temperatures.

Figure 2 displays C 1s XPS spectra collected while exposing Ni/CeO_2_(111) to a CH_4_/H_2_O mixture at 300–700 K. The pristine surface exhibits a broad feature from 293 to 288 eV attributed to the Ce 4s core level. This feature overlaps with the signal seen for the surface CO_*x*_ species formed by the full dissociation of methane and the reaction of carbon with surface oxygens.^27,28^ The interaction of the CH_4_/H_2_O gas mixture with Ni/CeO_2_(111) yields CO_*x*_, CH_*x*_O, and CH_*x*_ groups on the surface. The CH_*x*_O species were not observed when the Ni/CeO_2_(111) system was exposed to only methane.^27,28^ Therefore, they result from the direct reaction of OH and CH_*x*_ groups on the surface, pointing to an associative reaction pathway for the MSR process, which is in good agreement with the DFT results described in the next section. At 700 K, the CO_*x*_, CH_*x*_O, and CH_*x*_ species disappear from the catalyst surface. Thus, they are reaction intermediates that can be formed and removed easily, and no carbon deposition is observed on the catalyst surface.

**Figure 3:**
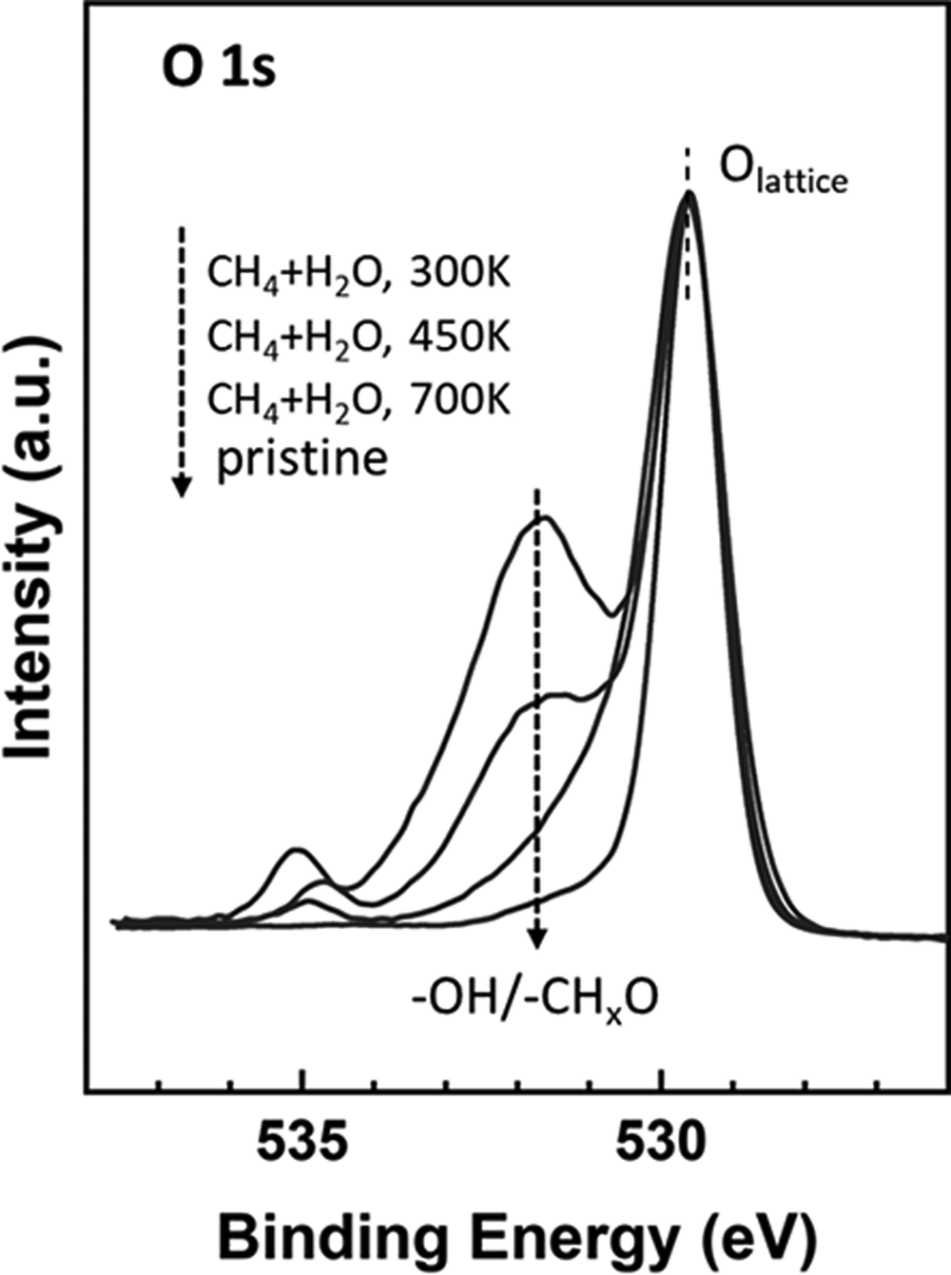
O 1s XPS spectra acquired while exposing a Ni/CeO_2_(111) surface (θ_Ni_ ∼ 0.15 ML) to a mixture of 25 mTorr of CH_4_ and 100 mTorr of H_2_O at the indicated temperatures.

An analysis of the O 1s XPS spectra collected under a gas mixture of CH_4_ and H_2_O shows interesting trends; see Figures 1 and 3. In the top traces of Figure 1, adding CH_4_ to H_2_O in the environment leads to an increase in the signal around 531.5–532 eV as a consequence of the formation of CH_*x*_O species on the Ni/CeO_2_(111) surface. The OH and CH_*x*_O species appear at similar binding energies in the O 1s region.^36^ In Figure 3, the signal for CH_*x*_O/OH is quite strong at 300 K, with the total coverage for the CH_*x*_O/OH groups being in the range of 0.6–0.8 ML. But these adsorbed species have limited stability, and their features decrease when the surface is heated to 450 K. At 700 K, CH_*x*_O is completely absent (Figure 2), and thus only a very small concentration of OH groups remains on the catalyst surface (Figures 1 and 3). The presence of these adsorbed OH groups is important because any CH_*x*_ species generated by methane dissociation can react with them to yield the products of the MSR process. Furthermore, Ni 2p and Ce 3d XPS spectra recorded under a mixture of methane and water (Figure S4) do not show any evidence for Ni^2+^ → Ni^0^ and Ce^4+^ → Ce^3+^ reductions, as seen in the case of pure methane (Figure S2). This is valid for all of the temperatures examined. Therefore, the ceria lattice oxygen is probably not involved in the MSR process on this catalyst, and the AP-XPS results support an associative mechanism that involves the formation of a CH_*x*_O intermediate, in agreement with the predictions of the DFT calculations discussed below.

### 3.2 Reaction Pathway of the MSR Reaction on Ni/CeO_2_(111): A DFT Study

**Figure 4:**
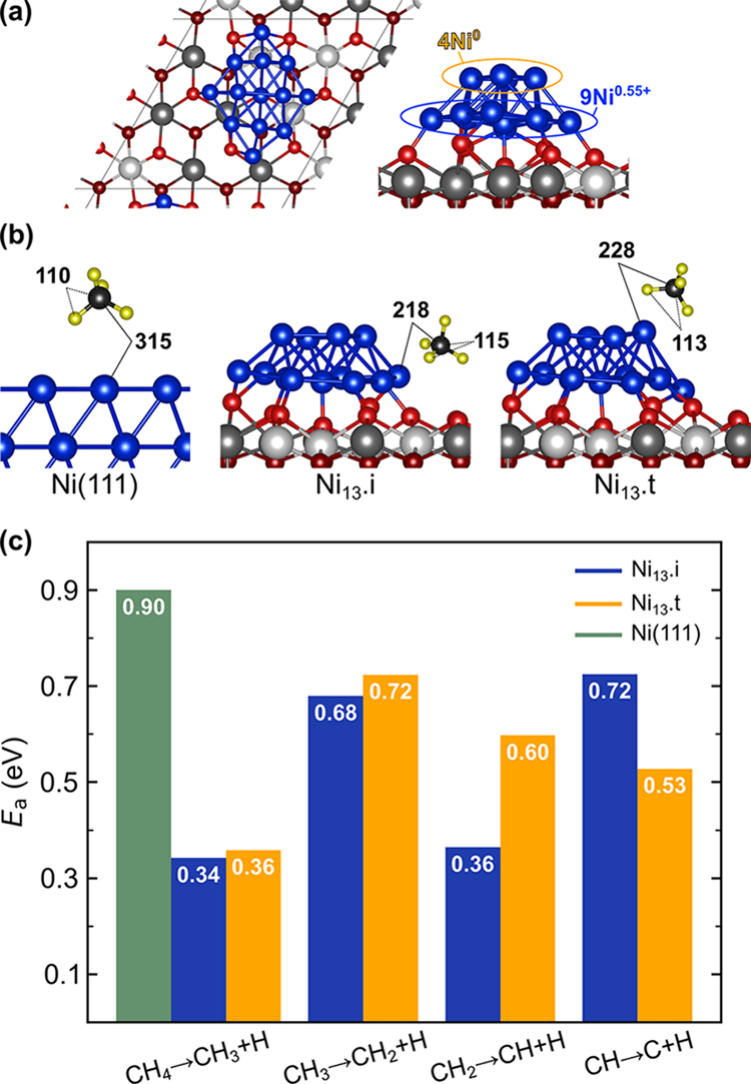
(a) Top and side views of the Ni_13_·CeO_2_ model catalyst surface. Surface/subsurface oxygen atoms in the outermost O–Ce–O trilayer are depicted in light/dark red, Ce^4+^/Ce^3+^ in light/dark gray, and Ni in blue. (b) Structure of the molecular adsorption of CH_4_ at Ni_13_·i and Ni_13_·t sites of the Ni_13_·CeO_2_ system, as well as on the Ni(111) surface.^30^ Selected interatomic distances (in pm) are indicated. (c) Activation energies (*E*_a_) for all CH_*x*_ dehydrogenation steps.

Using a Ni_13_ cluster supported on a flat CeO_2_(111) surface (Figures 4a and S1), we investigated the surface chemistry of the MSR process on Ni/CeO_2_. The Ni_13_ cluster reduces the ceria support upon adsorption with the formation of five Ce^3+^ ions. The calculated electronic structure of the Ni_13_·CeO_2_ system shows that the charge transfer by Ni atoms to the support is solely from the nine atoms in the interfacial layer, which are partially oxidized (9× Ni^0.55+^), whereas four neutral Ni atoms (4× Ni^0^) are above them (Table S1), in line with previous results.^34^ Hence, two types of Ni sites exist for adsorption and activation of reactants on the Ni_13_·CeO_2_ model catalyst, namely, oxidized interfacial sites and metallic terrace sites, hereafter referred to as Ni_13_·i and Ni_13_·t, respectively. The reaction pathway for methane steam reforming over the Ni_13_·CeO_2_ model catalysts is discussed below.

#### 3.2.1 CH_4_ Activation and Dehydrogenation

The cleavage of the first C–H bond through the dissociative adsorption of CH_4_ has generally been considered a rate-controlling step for the MSR reaction on Ni-based catalysts, based on the observed low reactivity of Ni surfaces toward methane^19^ and the high activation barrier for the CH_4_ → CH_3_ + H reaction on Ni(111) obtained in DFT studies.^25,26^ However, it was recently shown that methane activation occurs even at 300 K on small ceria-supported Ni particles,^27−30^ indicating much lower activation barriers than on the extended Ni surface. The first step in CH_4_ activation involves its molecular adsorption, which is very weak on the CeO_2_(111) surface,^74−77^ suggesting that methane should dissociate over Ni sites instead, as shown by XPS spectra of the pristine CeO_2_ and the Ni/CeO_2_ surfaces under 1 Torr of methane.^27,29^ Accordingly, we considered the adsorption and dehydrogenation of CH_4_ on Ni sites of the Ni_13_·CeO_2_ system.

CH_4_ adsorption on the Ni_13_ cluster is stronger by about 0.2 eV than that on the extended Ni(111) surface for both the interfacial Ni_13_·i and terrace Ni_13_·t sites (Figures S5, S7, and S8). Moreover, the CH_4_ molecule comes closer to the surface of the Ni cluster, with C–Ni distances of 218 (Ni_13_·i) and 228 pm (Ni_13_·t), compared to 315 pm on Ni(111)^30^ (Figure 4b). Inspection of the atom- and orbital-projected density of states (PDOS) onto the d-states of the Ni_13_·i and Ni_13_·t sites where CH_4_ adsorbs (Table S2) reveals that the d*_xz_* states become less occupied upon adsorption of the Ni_13_ cluster onto the ceria support. The consequence of such an effect is that the Pauli repulsion to the methane’s frontier orbital is reduced, enabling the molecule to come closer to the surface. The states are then occupied upon CH_4_ adsorption as measured by the decrease in the number of empty d*_xz_* states on both Ni_13_·i and Ni_13_·t sites in the CH_4_/Ni_13_·CeO_2_ system. As a result of the close approach of CH_4_, the C–H bond pointing toward the surface becomes preactivated, resulting in an increase in the bond length from 110 pm in the gas-phase CH_4_ molecule to 115 and 113 pm at the Ni_13_·i and Ni_13_·t sites, respectively (Figure 4b). Note that upon methane adsorption on the Ni(111) surface, the C–H bond is not stretched,^23,30^ and the occupation of d*_xz_* states remains unchanged (Table S3).

The first dehydrogenation step of these preactivated CH_4_ molecules takes place with low activation energy barriers of 0.34 eV at the Ni–CeO_2_ interface (Ni^0.55+^) and 0.36 eV at the Ni terrace (Ni^0^) (Figure 4c). We note that although interfacial Ni sites are partially oxidized and Ni atoms in the second layer of the cluster have a metallic character, the barriers are comparable. Hence, low-temperature CH_4_ activation on low-loaded Ni/CeO_2_ systems is expected to take place both at the perimeter of the Ni–CeO_2_ interface and on Ni atoms with no direct bonds to the support. The latter, however, is not the same as surface Ni atoms in Ni(111) with an activation barrier for the CH_4_ → CH_3_ + H reaction that is larger by 0.56 eV (0.90 eV, Figure S5). The combined effects of metal–support interactions and low metal loading contribute to the improved catalytic activity of Ni/CeO_2_ compared to Ni(111). Importantly, ceria-deposited small Ni clusters exhibit higher local fluxionality than Ni(111), i.e., Ni–Ni bonds are less rigid for the metal atoms in the clusters and can lead to stronger stabilizing interactions and lower activation energies on catalytic pathways (cf. the change in the average Ni–Ni bond length upon CH_4_ adsorption on Ni_13_·t, +11.7 pm, and on Ni(111), +0.2 pm; Table S4). Further dehydrogenation steps (CH_3_ → CH_2_ → CH → C) also proceed with relatively low barriers on the supported Ni_13_ cluster. The activation barriers (*E*_a_) for the elementary steps involved in CH_4_ dehydrogenation are shown in Figure 4c. The corresponding reaction energies (Δ*E*) and a comparison with previously published values for the Ni(111) surface are shown in Table S5, whereas the structures of the initial, final, and transition states are shown in Figures S7 and S8. The highest energy barrier at the Ni_13_·t sites corresponds to CH_3_ → CH_2_ + H dehydrogenation (0.72 eV), whereas at the Ni_13_·i sites, it is associated with CH → C + H dehydrogenation (0.72 eV). Similar to the above-discussed case of the first H abstraction from CH_4_, the comparison with the extended Ni(111) surface (Table S5) reveals that the last H abstraction from CH on Ni_13_·CeO_2_ has an activation barrier that is smaller by at least 0.6 eV than that on the extended surface, whereas the barriers for the second and third dehydrogenation steps in both systems are comparable.

The binding of isolated CH_*x*_ species (*x* = 0–3) on Ni_13_·CeO_2_ is stronger than that on Ni(111) (Table S6), with the largest difference of about 1 eV for the C atom. At both Ni_13_·i and Ni_13_·t sites, CH_3_ binds on a twofold bridge position and, although CH_2_ also binds on a bridge site upon its formation, it changes to a threefold site after the removal of the co-adsorbed H atoms (Figures S7 and S8). Note that on the extended Ni(111) surface, CH_3_ and CH_2_ adsorb on a threefold face-centered cubic (fcc) site.^53^ CH and C species bind to four Ni sites of the Ni_13_ cluster producing significant structural distortion (Figures S7 and S8), which might explain their higher stability compared to the more rigid Ni(111) surface. The fourfold binding is ascribed to the higher degree of unsaturation of the CH and C species, and it is also seen in the Ni(111) surface, where CH and C adsorb on hexagonal close-packed (hcp) hollow sites (instead of fcc), enabling their coordination with an additional Ni atom in the subsurface layer.^53^

In spite of the easy formation and increased stability of C atoms on Ni sites of the Ni_13_·CeO_2_ system, a low tendency toward carbon deposition is observed in the AP-XPS experiments performed over model Ni/CeO_2_ catalysts (cf. Figure 2), as well as in prior experimental studies.^28^ In this regard, it has been argued that carbon deposition on extended Ni surfaces depends strongly on the concentration of oxygen on the catalytic surface.^53^ In an oxygen-lacking environment, the interaction between CH_*x*_ intermediates and oxygen does not occur at a rate sufficient to convert the carbon produced from CH_4_ dehydrogenation to CO, thus resulting in carbon accumulation and subsequent deactivation of the catalysts.^53,54^ We show below that the Ni_13_·CeO_2_ surface provides unique sites and pathways suitable to convert carbon to CO in the MSR reaction with barriers below 0.9 eV.

#### 3.2.2 H_2_O Dissociative Adsorption

**Figure 5:**
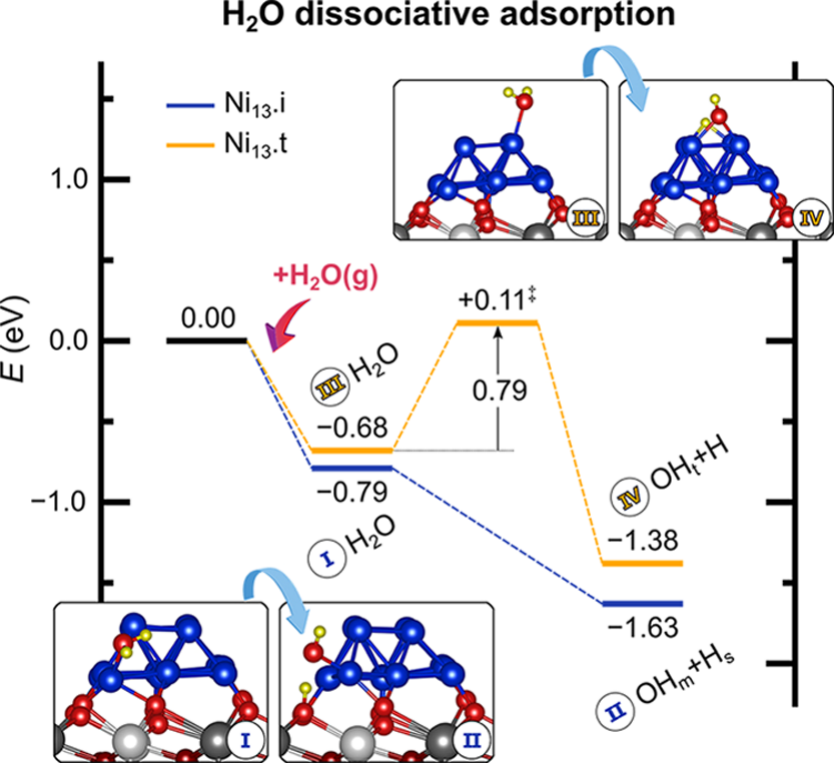
H_2_O dissociative adsorption on the Ni_13_·CeO_2_ surface. The yellow pathway describes the reaction over terrace sites of the Ni_13_ cluster, whereas the blue pathway shows the barrierless dissociation at the Ni–CeO_2_ interface. TSs are indicated by a double dagger ‡. Energies are referenced to the total energy of H_2_O_gas_ and the pristine Ni_13_·CeO_2_ surface.

H_2_O dissociates at the Ni–CeO_2_ interface through a virtually barrierless process, as previously shown for ceria-supported Ni single atoms and planar Ni_4_ clusters.^29,36^ The dissociative adsorption involves sites from both the Ni cluster and the CeO_2_ surface, with the OH group adsorbing monodentate (OH_m_) on Ni_13_·i and the dissociated proton on lattice oxygen from the ceria support (H_s_) (Figure 5). A hydrogen bond between OH_m_ and H_s_ is formed, stabilizing the structure (*d*(OH_m_–H_s_) = 179 pm). On the other hand, the dissociation of H_2_O on terrace sites of the Ni_13_ cluster does not involve lattice oxygen, producing a bidentate OH species on Ni_13_·t (OH_t_) and a H atom nearby on the cluster, and it is hindered by a barrier of 0.79 eV.

For comparison, the dissociative adsorption of water on Ni(111) is significantly less exothermic with Δ*E* = −0.41 eV,^24^ and it is hindered by a high barrier of 0.90–1.11 eV,^21,23,24,36^ whereas on the nondefective CeO_2_(111) surface, no true dissociation occurs and the molecular state coexists with an OH-pair-like configuration that easily recombines and desorbs at the reaction temperature.^36,78^ Therefore, these calculations show that H_2_O dissociates preferentially over the Ni–CeO_2_ interface, undergoing barrierless activation and easily producing adsorbed OH groups.

#### 3.2.3 CO Formation and Carbon Removal

**Figure 6:**
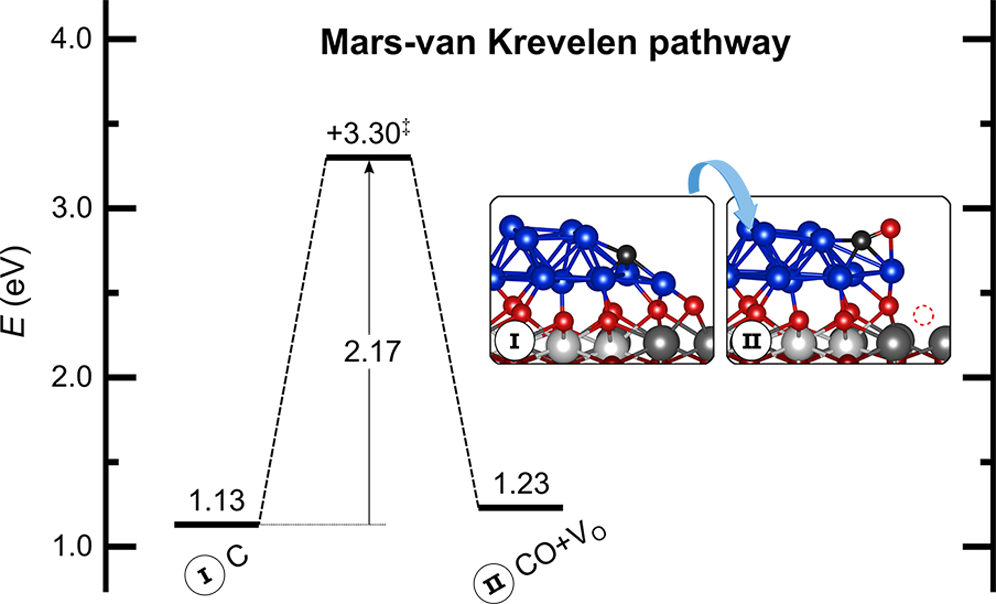
Formation of CO via a Mars–van Krevelen process involving the migration of lattice oxygen from the ceria surface to the Ni–CeO_2_ interface, leaving an oxygen vacancy. The TS is indicated by a double dagger ‡. Energies are referred to those of the clean surface and gas-phase species according to the stoichiometry of the MSR reaction.

Since chemisorbed CH_4_ on Ni/CeO_2_ easily loses all its hydrogens (Figure 4c), we first explore the oxidation of carbon on interfacial Ni_13_·i sites via its direct reaction with surface lattice oxygen (O_s_), resulting in a CO molecule adsorbed on the Ni cluster and an oxygen vacancy on the ceria support, which could later be reoxidized by water. This type of Mars–van Krevelen redox cycle has been suggested to be the route for many catalytic reactions involving CeO_2_.^79−81^ We note that C atoms adsorbed on terrace sites can easily migrate to the Ni–CeO_2_ interface with a barrier of 0.37 eV (Figure S8) and therefore they could be available for oxidation by lattice oxygen, even if CH_4_ activation and dehydrogenation take place on terrace sites. The formation of CO through the direct reaction of C with lattice oxygen has a very high barrier of 2.17 eV (Figure 6), and thus this pathway is deemed unlikely to take place. Instead, the adsorbed carbon atom could react with O or OH species chemisorbed on the Ni cluster to directly form CO from C + O or an oxidized COH intermediate that could then dehydrogenate to CO, which would be in line with the results of the AP-XPS study. Therefore, we investigated next the energy barriers involved in the formation of CO through these pathways.

**Figure 7:**
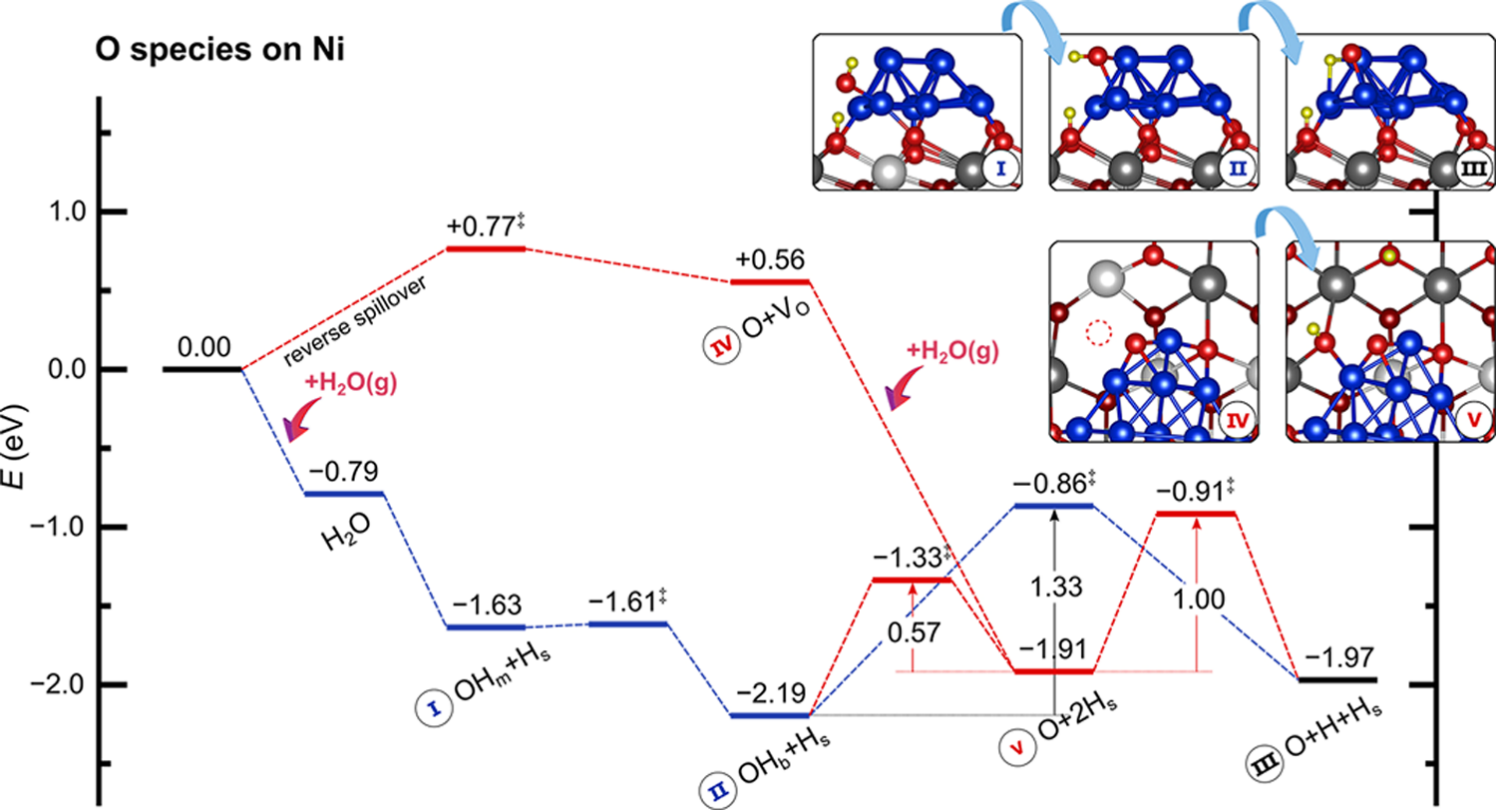
Formation of Ni–O species on the Ni_13_·CeO_2_ system. A pathway involving H_2_O dehydrogenation is shown in blue. The oxygen reverse spillover pathway is shown in red. H-diffusion steps have been omitted for simplicity. TS’s are indicated by a double dagger ‡. Energies are referenced to the total energy of H_2_O_gas_ and the pristine Ni_13_·CeO_2_ surface. H_s_ denotes H adsorbed on surface lattice oxygen (O_s_), OH_m_ and OH_b_ represent monodentate and bidentate binding at Ni_13_·i, respectively.

Regarding the existence of chemisorbed O species, as discussed above, H_2_O dissociates (OH_m_ + H_s_, Figure 5) at the Ni–CeO_2_ interface through a practically barrierless process. The monodentate OH_m_ species can migrate to a bidentate position OH_b_ (I → II in Figure 7, cf. Figure S9) to then dissociate into O and H species on the Ni cluster with a barrier of 1.33 eV (II → III), which is close to that reported for the Ni(111) surface (1.16–1.31 eV).^21,23,24^ We note that the possibility of forming OH and O species at interfacial Ni sites by migration of lattice O from the support to the Ni cluster (oxygen reverse spillover) has also been considered (red pathway in Figure 7). In this pathway, a surface lattice oxygen ion migrates to the cluster, leaving an oxygen vacancy on the CeO_2_(111) surface (IV), in an endothermic process with Δ*E* = 0.56 eV and *E*_a_ = 0.77 eV. Subsequently, H_2_O is activated at the oxygen vacancy site with no barrier,^82−85^ forming two H_s_ groups (V). The migration of H from the support to the Ni cluster involves a barrier of 1.00 eV to reach the O + H + H_s_ state (V → III). Alternatively, the H atom could bind to chemisorbed O with a barrier of 0.57 eV (V → II), resulting in an OH_b_ group on the Ni_13_ cluster. In summary, it is difficult to form O species chemisorbed on the Ni cluster and thus they are not easily available for the direct oxidation of C atoms. Moreover, the direct formation of CO from C and O atoms on the Ni cluster is hindered by a high barrier of 1.47 eV (cf. R7 in Table S7), further discouraging a pathway involving the direct oxidation of carbon with chemisorbed oxygen.

However, the reaction of C with OH groups readily available from the dissociation of H_2_O at the Ni–CeO_2_ interface produces the COH intermediate with an energy barrier of 0.89 eV, which is significantly lower than that of the abovementioned reaction of C with chemisorbed O (1.47 eV). This may be related to the significantly lower binding of the OH species compared to the O species (−3.97 and −5.86 eV, respectively; Table S6). The COH formation on the Ni_13_ cluster has also a lower barrier than those reported in the literature for Ni(111) (1.14–1.46 eV).^21,24,26^

**Figure 8:**
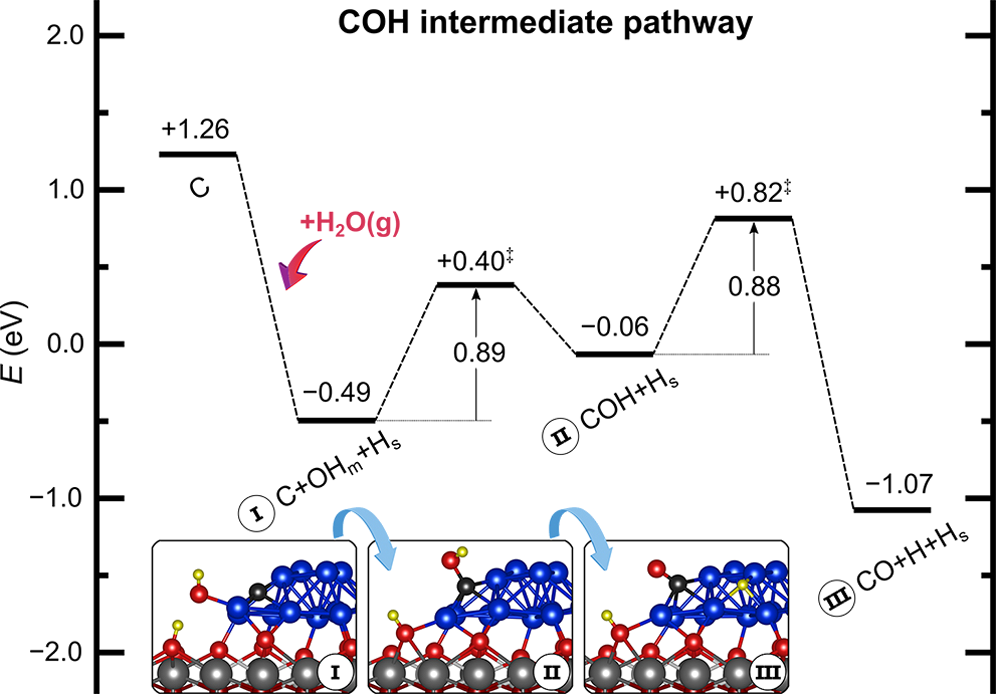
COH intermediate pathway for the MSR reaction over the Ni_13_·CeO_2_ system. TSs are indicated by a double dagger ‡. Energies of all states are referred to those of the clean surface and gas-phase species according to the stoichiometry of the MSR reaction.

Overall, these results allow us to propose a reaction pathway for the production of CO via the direct reaction of C with OH groups through a COH intermediate (Figure 8), and thus O species chemisorbed on the Ni cluster would not be required to oxidize carbon. The first step (I in Figure 8) corresponds to the barrierless activation of water at the Ni–CeO_2_ interface near a C atom on Ni_13_·i, with Δ*E* = −1.75 eV. Next, C and OH react to form the COH intermediate in an endothermic step (Δ*E* = 0.43 eV) with an energy barrier of 0.89 eV (I → II in Figure 8). Finally, a similar barrier of 0.88 eV must be overcome to dehydrogenate the COH intermediate and produce CO (II → III in Figure 8).

Structures and energies of all of the states involved in the COH intermediate pathway are detailed in Figure S10. Reaction and activation energies are summarized and compared with literature values for Ni(111) in Table S7. We note that the reaction of CH_*x*_ (*x* = 1–3) and OH to form CH_*x*_OH intermediates was also considered, but the barriers (*E*_a_ ≥ 0.81 eV) are larger than those of the dissociation of the CH_*x*_ species (*E*_a_ ≤ 0.72 eV), as shown in Figure S11. This indicates that CH_*x*_ could preferentially dehydrogenate fully to C and then react with OH, in line with the COH pathway presented above. However, it should be noted that adsorbate coverage effects can slow down the rate of CH_*x*_ dehydrogenation steps, particularly at low temperatures (≤450 K) at which a higher coverage of adsorbates (CH_*x*_, OH, H) is expected, reducing the availability of free active sites for the decomposition of methyl species. Thus, at such temperatures, various CH_*x*_ (*x* = 1–3) species can coexist on the catalyst surface (cf. Figure 2).

In a final step, CO and H_2_ must desorb to close the catalytic cycle of the endothermic MSR reaction. We observe that the XPS spectra do not show adsorbed CO (Figure 2); therefore, the desorption of CO should not be too difficult. It must be noted that gradient-corrected exchange–correlation functionals, such as PBE, overestimate the binding of CO on metal surfaces;^86−88^ therefore, desorption of the molecule is predicted to be more difficult than it actually is. For instance, the calculated CO adsorption on Ni(111) (Table S6) is overestimated by about 0.5 eV compared to the experimental value.^89^ On the other hand, H_2_ molecules can be easily formed from the bonding of two H species chemisorbed on the Ni_13_ cluster, with Δ*E* = 0.47 eV and a barrier *E*_a_ = 0.62 eV (Figure S12). As for the H species adsorbed on the surface lattice oxygen (H_s_), which are formed by water dissociation at the Ni–CeO_2_ interface, they would have to migrate from the support to the cluster before reacting with other H species to form H_2_. Direct migration is hindered by a high energy barrier of 1.48 eV, but the process becomes easier when assisted by additional water dissociated at the terrace sites of the Ni_13_ cluster, providing a pathway for which the highest barrier is *E*_a_ = 0.75 eV (Figure S12).

The results presented above reveal that the activation of CH_4_ and H_2_O and the formation of H_2_ occur with relatively small energy barriers of about 0.7 eV on Ni/CeO_2_, and the oxidation of carbon through an associative pathway involving the COH intermediate takes place with activation energy below 0.9 eV. This is quite different from the case of the extended Ni(111) surface, for which both the activation of methane and water and the oxidation of carbonaceous intermediates to form CO involve high energy barriers (≥1 eV, cf. Table S7).^21,25^ Furthermore, the barrierless activation of water at the Ni–CeO_2_ interface allows for a higher supply of OH species and, consequently, lower steam-to-methane ratios are required to achieve the same OH formation rate as that for extended Ni surfaces and traditional Ni catalysts supported on aluminum or magnesium oxides, for which H_2_O dissociation is not easy.

## 4 Conclusions

We conclude that the selectivity of the MSR reaction can be steered to prevent coke formation by choosing the “right” metal–oxide combination and controlling the effects of metal loading. Well-dispersed Ni nanoparticles supported on ceria are active and efficient MSR catalysts. The interactions between the reducible support and the small-sized nanoparticles are crucial for facile methane dehydrogenation and water dissociation at the Ni–CeO_2_ interface. Studies of AP-XPS indicate that CH_4_/H_2_O gas mixtures react with Ni/CeO_2_(111) surfaces to form OH, CH_*x*_, and CH_*x*_O at 300 K. All of these species are easily formed and desorb at temperatures below 700 K when the rate of the MSR process is accelerated. In line with the experiments, DFT calculations reveal a MSR reaction pathway with barriers below 1 eV that would enable reduced operating temperatures. The path proceeds via the formation of a COH intermediate species from chemisorbed C atoms and OH groups, hindering carbon accumulation and catalyst deactivation even with a low steam-to-methane ratio in the reactant feed. Water also facilitates the removal of hydrogen from the support at the Ni–CeO_2_ interface.

In summary, when undertaking the rational design and improvement of novel ceria-supported metal catalysts for the MSR reaction, it has to be taken into account that both CH_4_ and H_2_O activation steps occur very easily on low-loaded Ni/CeO_2_, and therefore the goal should be to modify the catalyst to decrease the barrier for the oxidation steps to form CO, for which one possibility may be to use Ni-based bimetallic catalysts.^24^ The properties of the ceria support material may also be chemically modified by, for example, doping with zirconium to improve its oxygen storage/transport characteristics that promote MSR pathways involving the participation of lattice oxygen, which was found to be unlikely in this study using pure ceria. This is in line with the recently observed promising performance of Ni catalysts supported on Zr-doped ceria for MSR at low temperatures.^14,90^ We anticipate that these strategies could represent an opportunity to further improve Ni/CeO_2_ catalysts and to guide the design of novel catalysts with lower kinetic barriers for the MSR reaction.

## Supporting Information Available

Ni 2p and Ce 3d spectra of AP-XPS for the interaction of methane, water, and methane/water mixtures with Ni/CeO_2_(111) catalysts; Ni_13_/CeO_2_(111) model catalyst surface; structures and energies of all initial states, transition states, and final states; selected projected density of states and Bader charge analysis (PDF)

## Notes

The DFT data that support the findings of this study are available in Materials Cloud Archive {https://www.materialscloud.org/home} with the identifier doi: 10.24435/materialscloud:ct-c6. The repository contains the calculations described in Figures S1 and S7–S12 in the Supporting Information. The data are also available from the authors upon reasonable request.

## References

[ref1] Rostrup-Nielsen J. R.; Christiansen L. J. Concepts in Syngas Manufacture; Imperial College Press: London, 2011.

[ref2] Jagannath A.; Elkamel A.; Karimi I. A. Improved Synthesis of Hydrogen Networks for Refineries. Ind. Eng. Chem. Res. 2014, 53, 16948–16963. 10.1021/ie5005042.

[ref3] Iulianelli A.; Liguori S.; Wilcox J.; Basile A. Advances on Methane Steam Reforming to Produce Hydrogen through Membrane Reactors Technology: A Review. Catal. Rev. 2016, 58, 1–35. 10.1080/01614940.2015.1099882.

[ref4] Oyama S. T.; Hacarlioglu P.; Gu Y.; Lee D. Dry Reforming of Methane Has No Future for Hydrogen Production: Comparison with Steam Reforming at High Pressure in Standard and Membrane Reactors. Int. J. Hydrogen Energy 2012, 37, 10444–10450. 10.1016/j.ijhydene.2011.09.149.

[ref5] Alves H. J.; Bley Junior C.; Niklevicz R. R.; Frigo E. P.; Frigo M. S.; Coimbra-Araújo C. H. Overview of Hydrogen Production Technologies from Biogas and the Applications in Fuel Cells. Int. J. Hydrogen Energy 2013, 38, 5215–5225. 10.1016/j.ijhydene.2013.02.057.

[ref6] Subramanian N.; Caravaca A.; García-García F. R.; Bowker M. Sustainable Hydrogen and/or Syngas Production: New Approaches to Reforming. Modern Developments in Catalysis; World Scientific: Singapore, 2017; pp 1–39.

[ref7] Lu J. B.; Wei G. H.; Zhu F. J.; Yan X. H.; Zhang J. L. Pressure Effect on the PEMFC Performance. Fuel Cells 2019, 19, 211–220. 10.1002/fuce.201800135.

[ref8] Matsumura Y.; Nakamori T. Steam Reforming of Methane over Nickel Catalysts at Low Reaction Temperature. Appl. Catal., A 2004, 258, 107–114. 10.1016/j.apcata.2003.08.009.

[ref9] Kusakabe K.; Sotowa K.; Eda T.; Iwamoto Y. Methane Steam Reforming over Ce–ZrO_2_-Supported Noble Metal Catalysts at Low Temperature. Fuel Process. Technol. 2004, 86, 319–326. 10.1016/j.fuproc.2004.05.003.

[ref10] Angeli S. D.; Monteleone G.; Giaconia A.; Lemonidou A. A. State-of-the-Art Catalysts for CH_4_ Steam Reforming at Low Temperature. Int. J. Hydrogen Energy 2014, 39, 1979–1997. 10.1016/j.ijhydene.2013.12.001.

[ref11] Rostrup-Nielsen J. R.; Sehested J.; Nørskov J. K. Hydrogen and Synthesis Gas by Steam- and CO_2_ Reforming. Adv. Catal. 2002, 47, 65–139. 10.1016/s0360-0564(02)47006-x.

[ref12] Sehested J. Four Challenges for Nickel Steam-Reforming Catalysts. Catal. Today 2006, 111, 103–110. 10.1016/j.cattod.2005.10.002.

[ref13] Iglesias I.; Baronetti G.; Mariño F. Ni/Ce_0.95_M_0.05_O_2–*d*_ (M = Zr, Pr, La) for Methane Steam Reforming at Mild Conditions. Int. J. Hydrogen Energy 2017, 42, 29735–29744. 10.1016/j.ijhydene.2017.09.176.

[ref14] Iglesias I.; Baronetti G.; Alemany L.; Mariño F. Insight into Ni/Ce_1–*x*_Zr_*x*_O_2−δ_ Support Interplay for Enhanced Methane Steam Reforming. Int. J. Hydrogen Energy 2019, 44, 3668–3680. 10.1016/j.ijhydene.2018.12.112.

[ref15] Wei J.; Iglesia E. Structural Requirements and Reaction Pathways in Methane Activation and Chemical Conversion Catalyzed by Rhodium. J. Catal. 2004, 225, 116–127. 10.1016/j.jcat.2003.09.030.

[ref16] Ross J. R. H.; Steel M. C. F. Mechanism of the Steam Reforming of Methane over a Coprecipitated Nickel-Alumina Catalyst. J. Chem. Soc., Faraday Trans. 1 1973, 69, 10 10.1039/f19736900010.

[ref17] Horn R.; Schlögl R. Methane Activation by Heterogeneous Catalysis. Catal. Lett. 2015, 145, 23–39. 10.1007/s10562-014-1417-z.

[ref18] Schwarz H.; Shaik S.; Li J. Electronic Effects on Room-Temperature, Gas-Phase C–H Bond Activations by Cluster Oxides and Metal Carbides: The Methane Challenge. J. Am. Chem. Soc. 2017, 139, 17201–17212. 10.1021/jacs.7b10139. 29112810

[ref19] Choudhary T. V.; Aksoylu E.; Wayne Goodman D. Nonoxidative Activation of Methane. Catal. Rev. 2003, 45, 151–203. 10.1081/CR-120017010.

[ref20] Xu J.; Froment G. F. Methane Steam Reforming, Methanation and Water-Gas Shift: I. Intrinsic Kinetics. AIChE J. 1989, 35, 88–96. 10.1002/aic.690350109.

[ref21] Zhu Y.-A.; Chen D.; Zhou X.-G.; Yuan W.-K. DFT Studies of Dry Reforming of Methane on Ni Catalyst. Catal. Today 2009, 148, 260–267. 10.1016/j.cattod.2009.08.022.

[ref22] Blaylock D. W.; Ogura T.; Green W. H.; Beran G. J. O. Computational Investigation of Thermochemistry and Kinetics of Steam Methane Reforming on Ni(111) under Realistic Conditions. J. Phys. Chem. C 2009, 113, 4898–4908. 10.1021/jp806527q.

[ref23] Han Z.; Yang Z.; Han M. Comprehensive Investigation of Methane Conversion over Ni(111) Surface under a Consistent DFT Framework: Implications for Anti-Coking of SOFC Anodes. Appl. Surf. Sci. 2019, 480, 243–255. 10.1016/j.apsusc.2019.02.084.

[ref24] Niu J.; Wang Y.; Qi Y.; Dam A. H.; Wang H.; Zhu Y.-A.; Holmen A.; Ran J.; Chen D. New Mechanism Insights into Methane Steam Reforming on Pt/Ni from DFT and Experimental Kinetic Study. Fuel 2020, 266, 117143 10.1016/j.fuel.2020.117143.

[ref25] Jones G.; Jakobsen J. G.; Shim S. S.; Kleis J.; Andersson M. P.; Rossmeisl J.; Abild-Pedersen F.; Bligaard T.; Helveg S.; Hinnemann B.; Rostrup-Nielsen J. R.; Chorkendorff I.; Sehested J.; Nørskov J. K. First Principles Calculations and Experimental Insight into Methane Steam Reforming over Transition Metal Catalysts. J. Catal. 2008, 259, 147–160. 10.1016/j.jcat.2008.08.003.

[ref26] Blaylock D. W.; Zhu Y. A.; Green W. H. Computational Investigation of the Thermochemistry and Kinetics of Steam Methane Reforming over a Multi-Faceted Nickel Catalyst. Top. Catal. 2011, 54, 828–844. 10.1007/s11244-011-9704-z.

[ref27] Lustemberg P. G.; Ramírez P. J.; Liu Z.; Gutiérrez R. A.; Grinter D. G.; Carrasco J.; Senanayake S. D.; Rodriguez J. A.; Ganduglia-Pirovano M. V. Room-Temperature Activation of Methane and Dry Re-Forming with CO_2_ on Ni-CeO_2_(111) Surfaces: Effect of Ce^3+^ Sites and Metal–Support Interactions on C–H Bond Cleavage. ACS Catal. 2016, 6, 8184–8191. 10.1021/acscatal.6b02360.

[ref28] Liu Z.; Grinter D. C.; Lustemberg P. G.; Nguyen-Phan T.-D.; Zhou Y.; Luo S.; Waluyo I.; Crumlin E. J.; Stacchiola D. J.; Zhou J.; Carrasco J.; Busnengo H. F.; Ganduglia-Pirovano M. V.; Senanayake S. D.; Rodriguez J. A. Dry Reforming of Methane on a Highly-Active Ni-CeO_2_ Catalyst: Effects of Metal-Support Interactions on C–H Bond Breaking. Angew. Chem., Int. Ed. 2016, 55, 7455–7459. 10.1002/anie.201602489. 27144344

[ref29] Lustemberg P. G.; Palomino R. M.; Gutiérrez R. A.; Grinter D. C.; Vorokhta M.; Liu Z.; Ramírez P. J.; Matolín V.; Ganduglia-Pirovano M. V.; Senanayake S. D.; Rodriguez J. A. Direct Conversion of Methane to Methanol on Ni-Ceria Surfaces: Metal-Support Interactions and Water-Enabled Catalytic Conversion by Site Blocking. J. Am. Chem. Soc. 2018, 140, 7681–7687. 10.1021/jacs.8b03809. 29804460

[ref30] Lustemberg P. G.; Zhang F.; Gutiérrez R. A.; Ramírez P. J.; Senanayake S. D.; Rodriguez J. A.; Ganduglia-Pirovano M. V. Breaking Simple Scaling Relations through Metal–Oxide Interactions: Understanding Room-Temperature Activation of Methane on M/CeO_2_ (M = Pt, Ni, or Co) Interfaces. J. Phys. Chem. Lett. 2020, 11, 9131–9137. 10.1021/acs.jpclett.0c02109. 33052684

[ref31] Lian Z.; Olanrele S. O.; Si C.; Yang M.; Li B. Critical Role of Interfacial Sites between Nickel and CeO_2_ Support in Dry Reforming of Methane: Revisit of Reaction Mechanism and Origin of Stability. J. Phys. Chem. C 2020, 124, 5118–5124. 10.1021/acs.jpcc.9b09856.

[ref32] Freund H.-J.; Pacchioni G. Oxide Ultra-Thin Films on Metals: New Materials for the Design of Supported Metal Catalysts. Chem. Soc. Rev. 2008, 37, 2224 10.1039/b718768h. 18818825

[ref33] Carrasco J.; Barrio L.; Liu P.; Rodriguez J. A.; Ganduglia-Pirovano M. V. Theoretical Studies of the Adsorption of CO and C on Ni(111) and Ni/CeO_2_(111): Evidence of a Strong Metal–Support Interaction. J. Phys. Chem. C 2013, 117, 8241–8250. 10.1021/jp400430r.

[ref34] Mao Z.; Lustemberg P. G.; Rumptz J. R.; Ganduglia-Pirovano M. V.; Campbell C. T. Ni Nanoparticles on CeO_2_(111): Energetics, Electron Transfer, and Structure by Ni Adsorption Calorimetry, Spectroscopies, and Density Functional Theory. ACS Catal. 2020, 10, 5101–5114. 10.1021/acscatal.0c00333.

[ref35] Ganduglia-Pirovano M. V. The Non-Innocent Role of Cerium Oxide in Heterogeneous Catalysis: A Theoretical Perspective. Catal. Today 2015, 253, 20–32. 10.1016/j.cattod.2015.01.049.

[ref36] Carrasco J.; López-Durán D.; Liu Z.; Duchoň T.; Evans J.; Senanayake S. D.; Crumlin E. J.; Matolín V.; Rodríguez J. A.; Ganduglia-Pirovano M. V. In Situ and Theoretical Studies for the Dissociation of Water on an Active Ni/CeO_2_ Catalyst: Importance of Strong Metal-Support Interactions for the Cleavage of O-H Bonds. Angew. Chem., Int. Ed. 2015, 54, 3917–3921. 10.1002/anie.201410697. 25651288

[ref37] Trovarelli A. Catalytic Properties of Ceria and CeO_2_-Containing Materials. Catal. Rev. 1996, 38, 439–520. 10.1080/01614949608006464.

[ref38] Conner W. C.; Falconer J. L. Spillover in Heterogeneous Catalysis. Chem. Rev. 1995, 95, 759–788. 10.1021/cr00035a014.

[ref39] Zafiris G. S.; Gorte R. J. Evidence for Low-Temperature Oxygen Migration from Ceria to Rh. J. Catal. 1993, 139, 561–567. 10.1006/jcat.1993.1049.

[ref40] Vayssilov G. N.; Lykhach Y.; Migani A.; Staudt T.; Petrova G. P.; Tsud N.; Skála T.; Bruix A.; Illas F.; Prince K. C.; Matolín V.; Neyman K. M.; Libuda J. Support Nanostructure Boosts Oxygen Transfer to Catalytically Active Platinum Nanoparticles. Nat. Mater. 2011, 10, 310–315. 10.1038/nmat2976. 21423188

[ref41] Ruiz Puigdollers A.; Schlexer P.; Tosoni S.; Pacchioni G. Increasing Oxide Reducibility: The Role of Metal/Oxide Interfaces in the Formation of Oxygen Vacancies. ACS Catal. 2017, 7, 6493–6513. 10.1021/acscatal.7b01913.

[ref42] Lykhach Y.; Kubát J.; Neitzel A.; Tsud N.; Vorokhta M.; Skála T.; Dvořák F.; Kosto Y.; Prince K. C.; Matolín V.; Johánek V.; Mysliveček J.; Libuda J. Charge Transfer and Spillover Phenomena in Ceria-Supported Iridium Catalysts: A Model Study. J. Chem. Phys. 2019, 151, 204703 10.1063/1.5126031. 31779319

[ref43] Ouyang M.; Boldrin P.; Maher R. C.; Chen X.; Liu X.; Cohen L. F.; Brandon N. P. A Mechanistic Study of the Interactions between Methane and Nickel Supported on Doped Ceria. Appl. Catal., B 2019, 248, 332–340. 10.1016/j.apcatb.2019.02.038.

[ref44] Roh H.-S.; Jun K.-W.; Dong W.-S.; Park S.-E.; Baek Y.-S. Highly Stable Ni Catalyst Supported on Ce–ZrO_2_ for Oxy-Steam Reforming of Methane. Catal. Lett. 2001, 74, 31–36. 10.1023/a:1016699317421.

[ref45] Damyanova S.; Pawelec B.; Palcheva R.; Karakirova Y.; Sanchez M. C. C.; Tyuliev G.; Gaigneaux E.; Fierro J. L. G. Structure and Surface Properties of Ceria-Modified Ni-Based Catalysts for Hydrogen Production. Appl. Catal., B 2018, 225, 340–353. 10.1016/j.apcatb.2017.12.002.

[ref46] Arcotumapathy V.; Alenazey F. S.; Al-Otaibi R. L.; Vo D.-V. N.; Alotaibi F. M.; Adesina A. A. Mechanistic Investigation of Methane Steam Reforming over Ce-Promoted Ni/SBA-15 Catalyst. Appl. Petrochem. Res. 2015, 5, 393–404. 10.1007/s13203-015-0121-2.

[ref47] Burghgraef H.; Jansen A. P. J.; van Santen R. A. Electronic Structure Calculations and Dynamics of Methane Activation on Nickel and Cobalt. J. Chem. Phys. 1994, 101, 11012–11020. 10.1063/1.467852.

[ref48] Burghgraef H.; Jansen A. P. J.; van Santen R. A. Methane Activation and Dehydrogenation on Nickel and Cobalt: A Computational Study. Surf. Sci. 1995, 324, 345–356. 10.1016/0039-6028(94)00716-0.

[ref49] Kratzer P.; Hammer B.; Nørskov J. K. A Theoretical Study of CH_4_ Dissociation on Pure and Gold-alloyed Ni(111) Surfaces. J. Chem. Phys. 1996, 105, 5595–5604. 10.1063/1.472399.

[ref50] Watwe R. M.; Bengaard H. S.; Rostrup-Nielsen J. R.; Dumesic J. A.; Nørskov J. K. Theoretical Studies of Stability and Reactivity of CH_*x*_ Species on Ni(111). J. Catal. 2000, 189, 16–30. 10.1006/jcat.1999.2699.

[ref51] Bengaard H. S.; Nørskov J. K.; Sehested J.; Clausen B. S.; Nielsen L. P.; Molenbroek A. M.; Rostrup-Nielsen J. R. Steam Reforming and Graphite Formation on Ni Catalysts. J. Catal. 2002, 209, 365–384. 10.1006/jcat.2002.3579.

[ref52] Abild-Pedersen F.; Greeley J.; Nørskov J. K. Understanding the Effect of Steps, Strain, Poisons, and Alloying: Methane Activation on Ni Surfaces. Catal. Lett. 2005, 105, 9–13. 10.1007/s10562-005-7998-9.

[ref53] Wang S.-G.; Cao D.-B.; Li Y.-W.; Wang J.; Jiao H. CO_2_ Reforming of CH_4_ on Ni(111): A Density Functional Theory Calculation. J. Phys. Chem. B 2006, 110, 9976–9983. 10.1021/jp060992g. 16706455

[ref54] Wang S.-G.; Liao X.-Y.; Hu J.; Cao D.-B.; Li Y.-W.; Wang J.; Jiao H. Kinetic Aspect of CO_2_ Reforming of CH_4_ on Ni(111): A Density Functional Theory Calculation. Surf. Sci. 2007, 601, 1271–1284. 10.1016/j.susc.2006.12.059.

[ref55] Kresse G.; Hafner J. Ab Initio Molecular Dynamics for Liquid Metals. Phys. Rev. B 1993, 47, 558–561. 10.1103/PhysRevB.47.558. 10004490

[ref56] Kresse G.; Furthmüller J. Efficient Iterative Schemes for Ab Initio Total-Energy Calculations Using a Plane-Wave Basis Set. Phys. Rev. B 1996, 54, 11169–11186. 10.1103/PhysRevB.54.11169. 9984901

[ref57] Perdew J. P.; Burke K.; Ernzerhof M. Generalized Gradient Approximation Made Simple. Phys. Rev. Lett. 1996, 77, 3865–3868. 10.1103/PhysRevLett.77.3865. 10062328

[ref58] Blöchl P. E. Projector Augmented-Wave Method. Phys. Rev. B 1994, 50, 17953–17979. 10.1103/PhysRevB.50.17953. 9976227

[ref59] Kresse G.; Joubert D. From Ultrasoft Pseudopotentials to the Projector Augmented-Wave Method. Phys. Rev. B 1999, 59, 1758–1775. 10.1103/PhysRevB.59.1758.

[ref60] Dudarev S. L.; Botton G. A.; Savrasov S. Y.; Humphreys C. J.; Sutton A. P. Electron-Energy-Loss Spectra and the Structural Stability of Nickel Oxide: An LSDA+U Study. Phys. Rev. B 1998, 57, 1505–1509. 10.1103/PhysRevB.57.1505.

[ref61] Nolan M.; Parker S. C.; Watson G. W. The Electronic Structure of Oxygen Vacancy Defects at the Low Index Surfaces of Ceria. Surf. Sci. 2005, 595, 223–232. 10.1016/j.susc.2005.08.015.

[ref62] Ganduglia-Pirovano M. V.; Hofmann A.; Sauer J. Oxygen Vacancies in Transition Metal and Rare Earth Oxides: Current State of Understanding and Remaining Challenges. Surf. Sci. Rep. 2007, 62, 219–270. 10.1016/j.surfrep.2007.03.002.

[ref63] Loschen C.; Carrasco J.; Neyman K. M.; Illas F. First-Principles LDA+U and GGA+*U* Study of Cerium Oxides: Dependence on the Effective U Parameter. Phys. Rev. B: Condens. Matter Mater. Phys. 2007, 75, 1–8. 10.1103/PhysRevB.75.035115.

[ref64] Skorodumova N. V.; Simak S. I.; Lundqvist B. I.; Abrikosov I. A.; Johansson B. Quantum Origin of the Oxygen Storage Capability of Ceria. Phys. Rev. Lett. 2002, 89, 166601 10.1103/PhysRevLett.89.166601. 12398742

[ref65] Ganduglia-Pirovano M. V.; Da Silva J. L. F.; Sauer J. Density-Functional Calculations of the Structure of Near-Surface Oxygen Vacancies and Electron Localization on CeO_2_(111). Phys. Rev. Lett. 2009, 102, 026101 10.1103/PhysRevLett.102.026101. 19257295

[ref66] Fabris S.; Vicario G.; Balducci G.; de Gironcoli S.; Baroni S. Electronic and Atomistic Structures of Clean and Reduced Ceria Surfaces. J. Phys. Chem. B 2005, 109, 22860–22867. 10.1021/jp0511698. 16853978

[ref67] Cococcioni M.; de Gironcoli S. Linear Response Approach to the Calculation of the Effective Interaction Parameters in the LDA+U Method. Phys. Rev. B 2005, 71, 035105 10.1103/PhysRevB.71.035105.

[ref68] Grimme S.; Antony J.; Ehrlich S.; Krieg H. A Consistent and Accurate Ab Initio Parametrization of Density Functional Dispersion Correction (DFT-D) for the 94 Elements H-Pu. J. Chem. Phys. 2010, 132, 154104 10.1063/1.3382344. 20423165

[ref69] Grimme S.; Ehrlich S.; Goerigk L. Effect of the Damping Function in Dispersion Corrected Density Functional Theory. J. Comput. Chem. 2011, 32, 1456–1465. 10.1002/jcc.21759. 21370243

[ref70] Bader R. F. W. A Quantum Theory of Molecular Structure and Its Applications. Chem. Rev. 1991, 91, 893–928. 10.1021/cr00005a013.

[ref71] Henkelman G.; Arnaldsson A.; Jónsson H. A Fast and Robust Algorithm for Bader Decomposition of Charge Density. Comput. Mater. Sci. 2006, 36, 354–360. 10.1016/j.commatsci.2005.04.010.

[ref72] Monkhorst H. J.; Pack J. D. Special Points for Brillouin-Zone Integrations. Phys. Rev. B 1976, 13, 5188–5192. 10.1103/PhysRevB.13.5188.

[ref73] Henkelman G.; Uberuaga B. P.; Jónsson H. A Climbing Image Nudged Elastic Band Method for Finding Saddle Points and Minimum Energy Paths. J. Chem. Phys. 2000, 113, 9901–9904. 10.1063/1.1329672.

[ref74] Knapp D.; Ziegler T. Methane Dissociation on the Ceria (111) Surface. J. Phys. Chem. C 2008, 112, 17311–17318. 10.1021/jp8039862.

[ref75] Krcha M. D.; Mayernick A. D.; Janik M. J. Periodic Trends of Oxygen Vacancy Formation and C–H Bond Activation over Transition Metal-Doped CeO_2_(111) Surfaces. J. Catal. 2012, 293, 103–115. 10.1016/j.jcat.2012.06.010.

[ref76] Fronzi M.; Piccinin S.; Delley B.; Traversa E.; Stampfl C. CH_*x*_ Adsorption (x = 1–4) and Thermodynamic Stability on the CeO_2_(111) Surface: A First-Principles Investigation. RSC Adv. 2014, 4, 12245 10.1039/c4ra01224k.

[ref77] Salcedo A.; Iglesias I.; Mariño F.; Irigoyen B. Promoted Methane Activation on Doped Ceria via Occupation of Pr(4f) States. Appl. Surf. Sci. 2018, 458, 397–404. 10.1016/j.apsusc.2018.07.090.

[ref78] Fernández-Torre D.; Kośmider K.; Carrasco J.; Ganduglia-Pirovano M. V.; Pérez R. Insight into the Adsorption of Water on the Clean CeO_2_(111) Surface with van Der Waals and Hybrid Density Functionals. J. Phys. Chem. C 2012, 116, 13584–13593. 10.1021/jp212605g.

[ref79] Kurungot S.; Yamaguchi T. Stability Improvement of Rh/γ-Al_2_O_3_ Catalyst Layer by Ceria Doping for Steam Reforming in an Integrated Catalytic Membrane Reactor System. Catal. Lett. 2004, 92, 181–187. 10.1023/B:CATL.0000014343.67377.98.

[ref80] Huang M.; Fabris S. CO Adsorption and Oxidation on Ceria Surfaces from DFT+U Calculations. J. Phys. Chem. C 2008, 112, 8643–8648. 10.1021/jp709898r.

[ref81] Vohs J. M. Site Requirements for the Adsorption and Reaction of Oxygenates on Metal Oxide Surfaces. Chem. Rev. 2013, 113, 4136–4163. 10.1021/cr300328u. 23181433

[ref82] Chen H.-T.; Choi Y. M.; Liu M.; Lin M. C. A Theoretical Study of Surface Reduction Mechanisms of CeO_2_(111) and (110) by H_2_. ChemPhysChem 2007, 8, 849–855. 10.1002/cphc.200600598. 17377938

[ref83] Yang Z.; Wang Q.; Wei S.; Ma D.; Sun Q. The Effect of Environment on the Reaction of Water on the Ceria(111) Surface: A DFT+U Study. J. Phys. Chem. C 2010, 114, 14891–14899. 10.1021/jp101057a.

[ref84] Marrocchelli D.; Yildiz B. First-Principles Assessment of H_2_S and H_2_O Reaction Mechanisms and the Subsequent Hydrogen Absorption on the CeO_2_(111) Surface. J. Phys. Chem. C 2012, 116, 2411–2424. 10.1021/jp205573v.

[ref85] Salcedo A.; Irigoyen B. Unraveling the Origin of Ceria Activity in Water–Gas Shift by First-Principles Microkinetic Modeling. J. Phys. Chem. C 2020, 124, 7823–7834. 10.1021/acs.jpcc.0c00229.

[ref86] Abild-Pedersen F.; Andersson M. P. CO Adsorption Energies on Metals with Correction for High Coordination Adsorption Sites – A Density Functional Study. Surf. Sci. 2007, 601, 1747–1753. 10.1016/j.susc.2007.01.052.

[ref87] Schimka L.; Harl J.; Stroppa A.; Grüneis A.; Marsman M.; Mittendorfer F.; Kresse G. Accurate Surface and Adsorption Energies from Many-Body Perturbation Theory. Nat. Mater. 2010, 9, 741–744. 10.1038/nmat2806. 20657589

[ref88] Patra A.; Peng H.; Sun J.; Perdew J. P. Rethinking CO Adsorption on Transition-Metal Surfaces: Effect of Density-Driven Self-Interaction Errors. Phys. Rev. B 2019, 100, 035442 10.1103/PhysRevB.100.035442.

[ref89] Stuckless J. T.; Al-Sarraf N.; Wartnaby C.; King D. A. Calorimetric Heats of Adsorption for CO on Nickel Single Crystal Surfaces. J. Chem. Phys. 1993, 99, 2202–2212. 10.1063/1.465282.

[ref90] Iglesias I.; Forti M.; Baronetti G.; Mariño F. Zr-Enhanced Stability of Ceria Based Supports for Methane Steam Reforming at Severe Reaction Conditions. Int. J. Hydrogen Energy 2019, 44, 8121–8132. 10.1016/j.ijhydene.2019.02.070.

