# Supplementary material for: Reaction Pathway for Coke-Free Methane Steam Reforming on a Ni/CeO2 Catalyst: Active Sites and the Role of Metal–Support Interactions
Source: ACS Catal. 2021 Jun 23;11(13):8327–37. doi: 10.1021/acscatal.1c01604 (PMC8294006; doi:10.1021/acscatal.1c01604)
Supplement: Supplementary file 1 — cs1c01604_si_001.pdf [file cs1c01604_si_001.pdf]

SUPPORTING INFORMATION

Reaction Pathway for Coke-Free Methane Steam Reforming on a Ni/CeO_2 Catalyst: Active Sites and the Role of Metal–Support Interactions

Agustín Salcedo[1,2], Pablo G. Lustemberg[3,4], Ning Rui[5], Robert M. Palomino[5], Zongyuan Liu[5], Slavomir Nemsak[6], Sanjaya D. Senanayake[5], José A. Rodriguez*[5], M. Verónica Ganduglia-Pirovano[*3], Beatriz Irigoyen[*1,2]

[1]Departamento de Ingeniería Química, Facultad de Ingeniería, Universidad de Buenos Aires (UBA), Ciudad Universitaria, C1428EGA Buenos Aires, Argentina

[2]Instituto de Tecnologías del Hidrógeno y Energías Sostenibles (ITHES, CONICET-UBA), Ciudad Universitaria, C1428EGA Buenos Aires, Argentina

[3]Instituto de Catálisis y Petroleoquímica (ICP, CSIC), 28049 Madrid, Spain

[4]Instituto de Física Rosario (IFIR, CONICET-UNR), S2000EKF Rosario, Santa Fe, Argentina

[5]Chemistry Division, Brookhaven National Laboratory, Upton, New York 11973, United States

[6]Advanced Light Source, Lawrence Berkeley National Laboratory, Berkeley, California 94720, United States

Corresponding authors:

*beatriz@di.fcen.uba.ar

*vgp@icp.csic.es

*rodrigez@bnl.gov

$Ni_{13}/CeO_2(111)$ Model

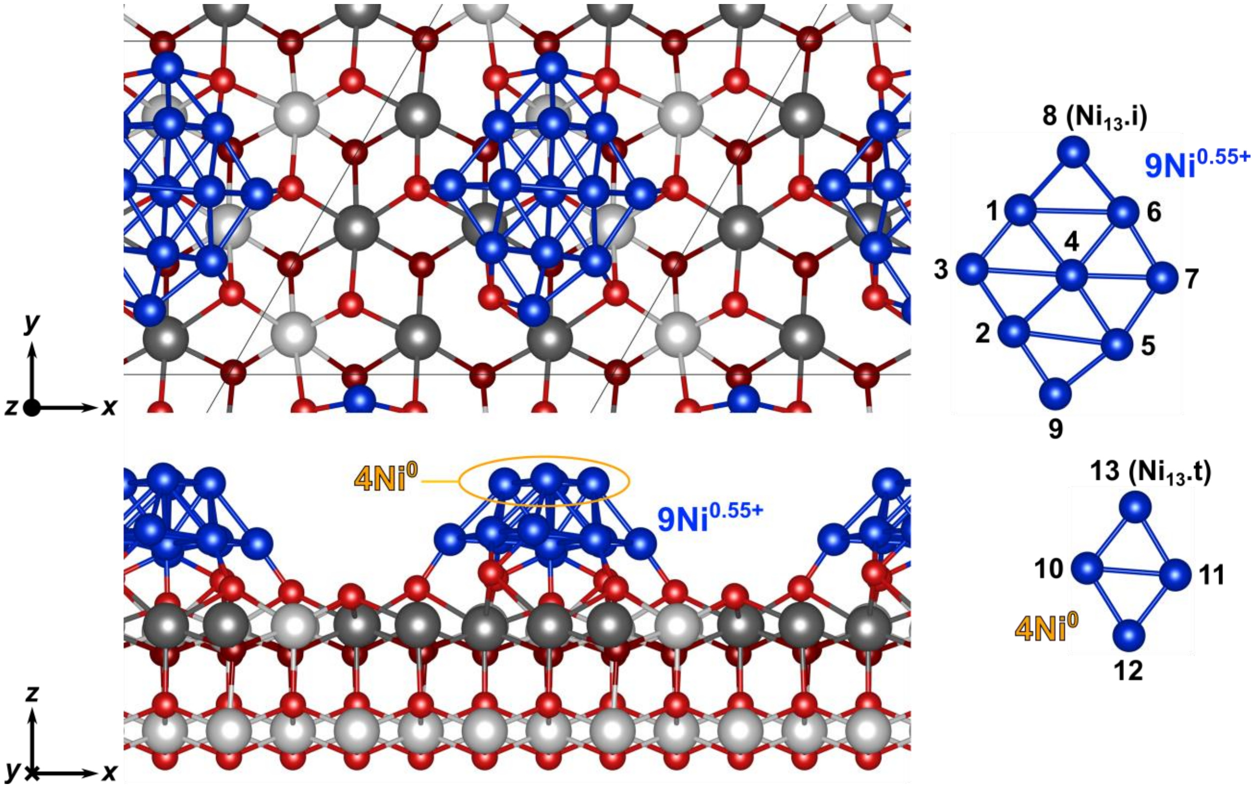

Figure S1. Top and side views of the $Ni_{13}/CeO_2(111)$ model catalyst surface, referred as $Ni_{13}.CeO_2$. The cartesian axes of the slab calculation are shown on the left. Surface/subsurface oxygen atoms in the outermost O−Ce−O trilayer are depicted in light/dark red, Ce^{4+}/Ce^{3+} in light/dark gray, and Ni in blue. The Ni sites at the Ni-CeO_2 interface (#8, Ni_{13}.i) and at the terrace of the Ni_{13} cluster (#13, Ni_{13}.t) where CH_4 is adsorbed are indicated.

Ni 2p and Ce 3d XPS Spectra

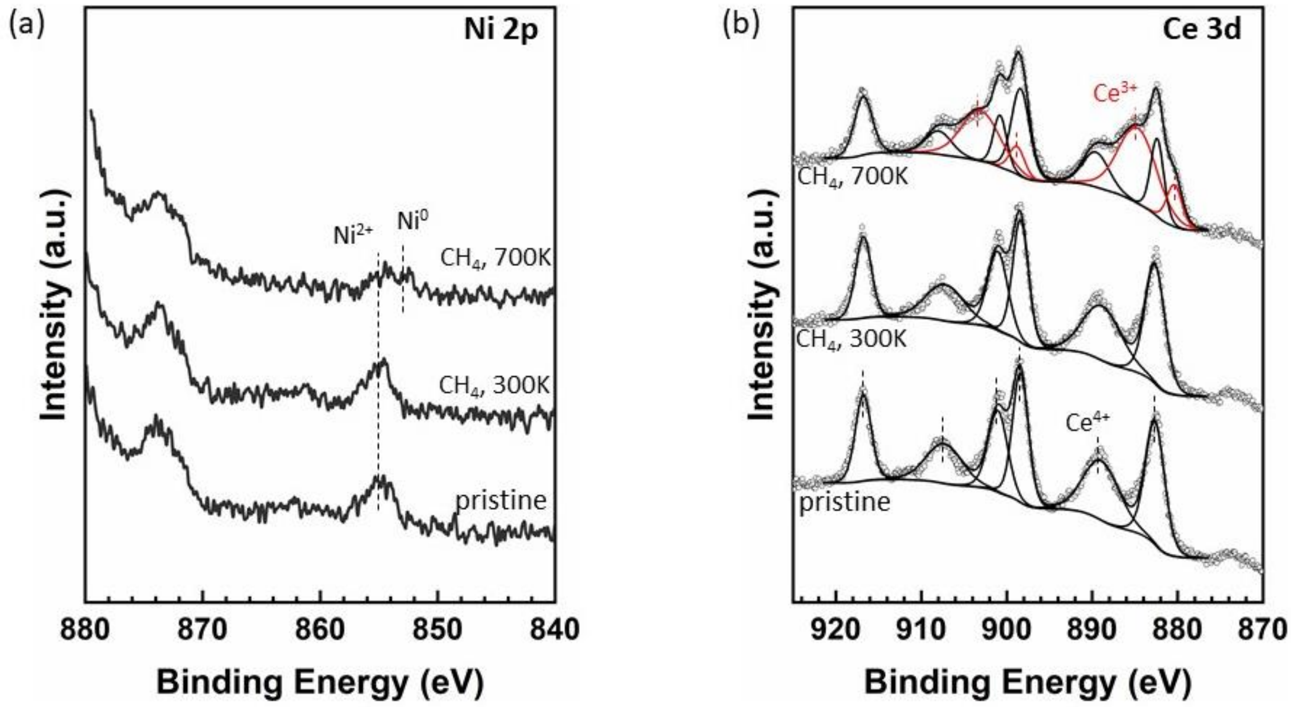

Figure S2. Ni 2p and Ce 3d XPS spectra collected while exposing a Ni/CeO_2(111) surface (θ_{Ni} ~ 0.15 ML) to 20 mTorr of methane at different temperatures. At 700 K, there is a coexistence of Ce^{4+} and Ce^{3+} cations in the oxide support as shown by curve-fitting the corresponding Ce 3d XPS spectrum.

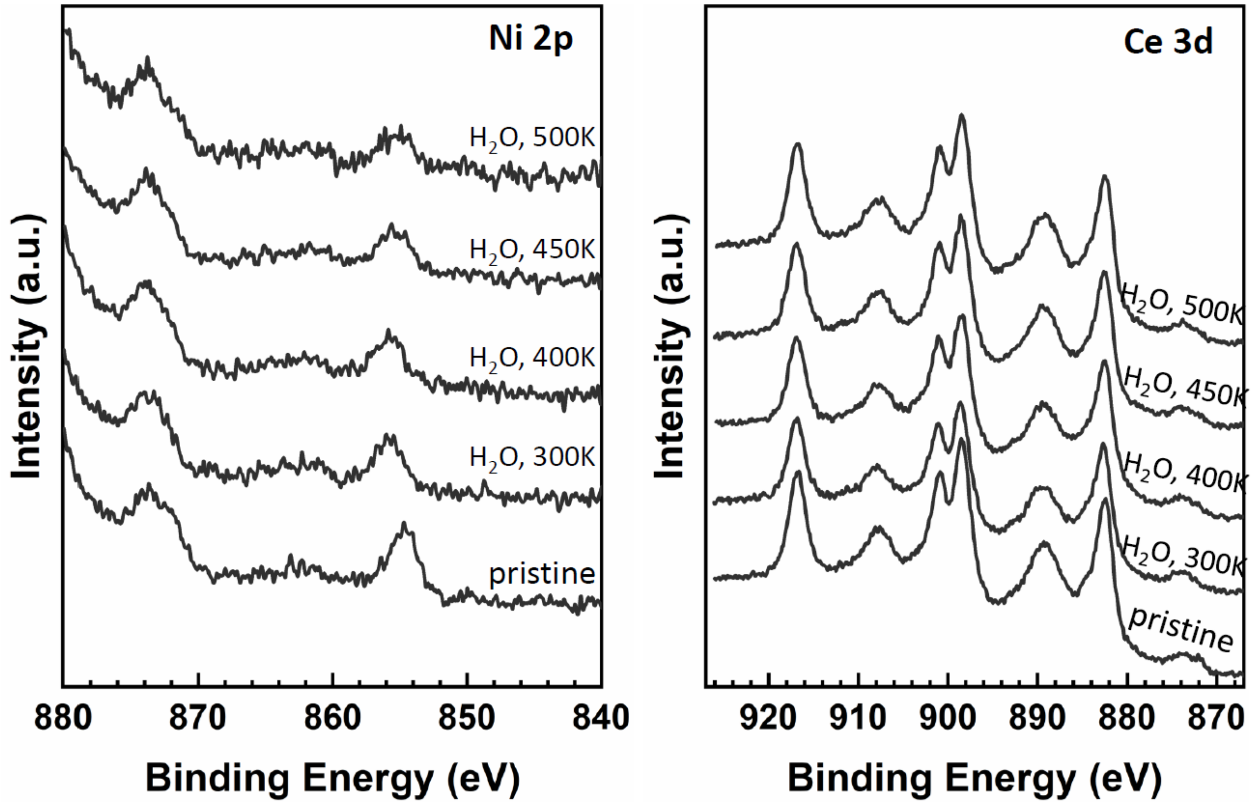

Figure S3. Ni 2p and Ce 3d XPS spectra collected while exposing a Ni/CeO_2(111) surface (θ_{Ni} ~ 0.15 ML) to 40 mTorr of water at different temperatures.

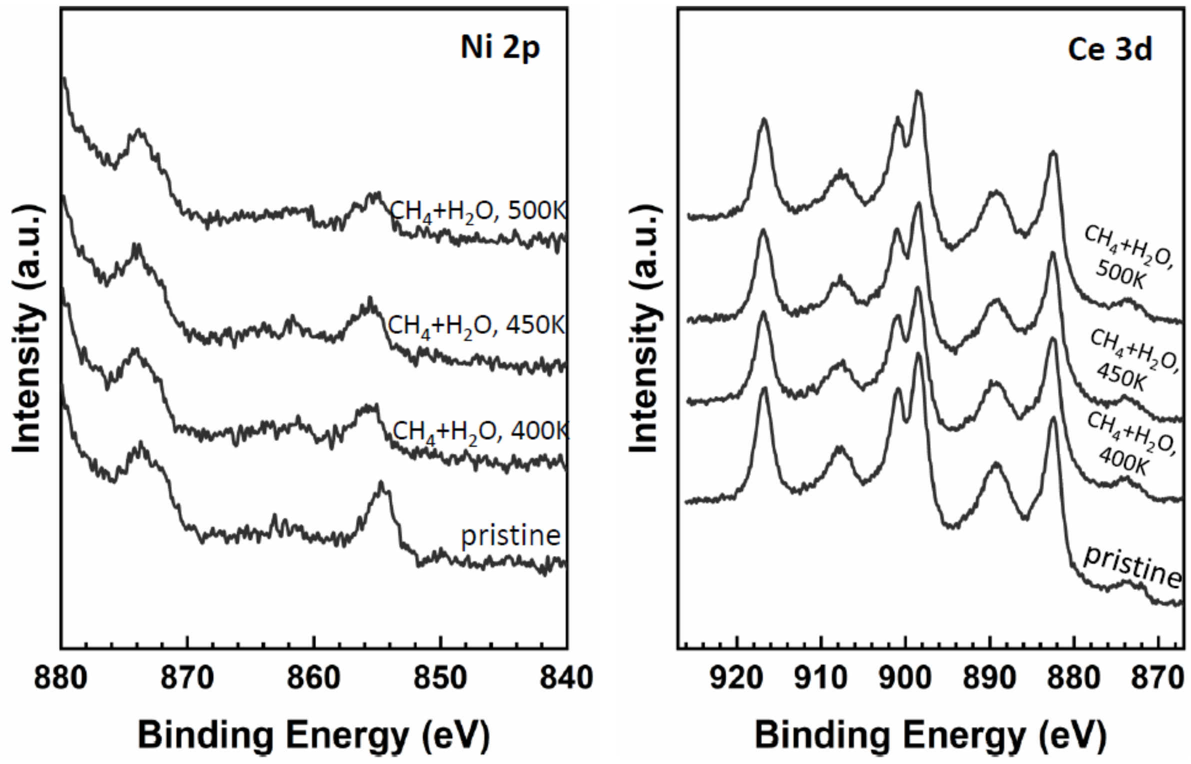

Figure S4. Ni 2p and Ce 3d XPS spectra collected while exposing a Ni/CeO_2(111) surface (θ_{Ni} ~ 0.15 ML) to 20 mTorr of methane and 40 mTorr of water at different temperatures.

Table S1. Bader charges (in |e|) for the Ni atoms of the Ni_{13} cluster (cf. Figure S1) before (Ni_{13}.gas) and after (Ni_{13}.CeO_2) its adsorption on the CeO_2(111) surface. Ni_{13}.gas corresponds to the gas-phase cluster resulting from the removal of the CeO_2 support from the Ni_{13}.CeO_2 system, without further geometry optimization. For Ni, 16 electrons ($3p^6 3d^8 4s^2$) were considered as valence. The difference in Bader charge upon adsorption is also listed.

# Ni atom	Ni_{13}.gas	Ni_{13}.CeO_2	Ni_{13}.gas−Ni_{13}.CeO_2
**1**	15.9926	15.7760	−0.22
**2**	15.9945	15.7686	−0.23
**3**	16.0759	15.7584	−0.32
**4**	15.8241	15.6206	−0.20
**5**	15.9925	15.7271	−0.27
**6**	15.9845	15.7570	−0.23
**7**	16.0961	15.7783	−0.32
**8**	16.0833	15.4933	−0.59
**9**	16.0713	15.7255	−0.35
**10**	15.9764	16.0614	0.08
**11**	15.9647	16.0312	0.07
**12**	15.9785	16.0361	0.06
**13**	15.9656	15.9869	0.02
**Total**	208.0000	205.5204	−2.48

Table S2. Integration of the projected density of states onto the d-states of a Ni atom at the interface (Ni_{13}.i) and on the terrace (Ni_{13}.t) of the Ni_{13} cluster (cf. Figure S1), within the 0 (E_F) to +1.0 eV energy interval. The principal axes for the *lm* decomposition are the cartesian axes of the calculation slab. The *z*-axis is aligned with the direction perpendicular to the surface, whereas the *x*- and *y*- axes are parallel to the surface. Ni_{13}.gas corresponds to the gas-phase cluster resulting from the removal of the CeO_2 support from the Ni_{13}.CeO_2 system, without further geometry optimization. CH_4/Ni_{13}.CeO_2 corresponds to the CH_4 adsorption on Ni_{13}.CeO_2.

Site	Band	Empty states			Difference of empty states	
		Ni_{13}.gas	Ni_{13}.CeO_2	CH_4/Ni_{13}.CeO_2	Ni_{13}.CeO_2−Ni_{13}.gas	CH_4/Ni_{13}.CeO_2−Ni_{13}.CeO_2
**Ni_{13}.t**	**dxy**	0.19	0.21	0.16	0.02	−0.05
	**dyz**	0.15	0.12	0.07	−0.04	−0.04
	**dz^2**	0.07	0.04	0.12	−0.03	0.09
	**dxz**	0.30	0.35	0.23	0.05	−0.12
	**$dx^2−y^2$**	0.19	0.10	0.09	−0.09	−0.01
	**dtotal**	0.90	0.81	0.67	−0.09	−0.14
**Ni_{13}.i**	**dxy**	0.23	0.16	0.28	−0.08	0.12
	**dyz**	0.24	0.06	0.13	−0.18	0.07
	**dz^2**	0.14	0.06	0.21	−0.08	0.15
	**dxz**	0.12	0.39	0.10	0.27	−0.29
	**$dx^2−y^2$**	0.10	0.13	0.03	0.03	−0.10
	**dtotal**	0.83	0.79	0.75	−0.04	−0.04

CH_4 Activation on the Ni(111) Surface

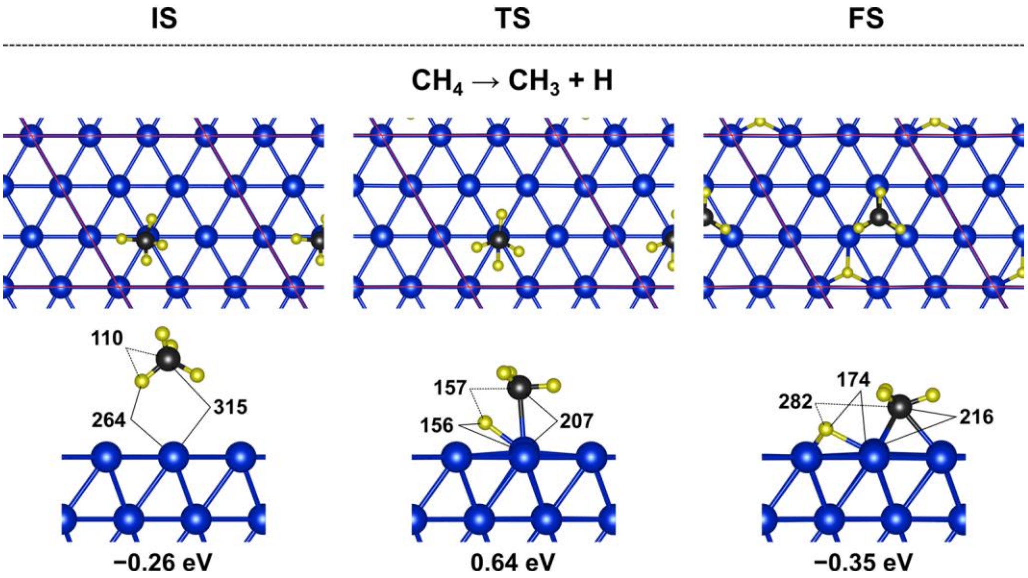

Figure S5. Structure and energy (relative to CH_4 in the gas phase and the pristine surface) of the initial state (IS), transition state (TS), and final state (FS) for the first dehydrogenation of CH_4 on the Ni(111) surface.[1] Selected interatomic distances (in pm) are indicated.

Table S3. Integration of the projected density of states onto the d-states of the Ni atom of the Ni(111) surface where CH_4 is adsorbed, within the 0 (E_F) to +1.0 eV energy interval. The principal axes for the *lm* decomposition are the cartesian axes of the calculation slab. The *z*-axis is aligned with the direction perpendicular to the surface, whereas the *x*- and *y*-axes are parallel to the surface.

Band	Empty states		Difference of empty states
	Ni(111)	CH_4/Ni(111)	CH_4/Ni(111)−Ni(111)
**dxy**	0.172	0.171	−0.001
**dyz**	0.119	0.116	−0.003
**dz^2**	0.183	0.202	0.018
**dxz**	0.119	0.117	−0.003
**$dx^2{-}y^2$**	0.172	0.171	−0.001
**dtotal**	0.765	0.776	0.011

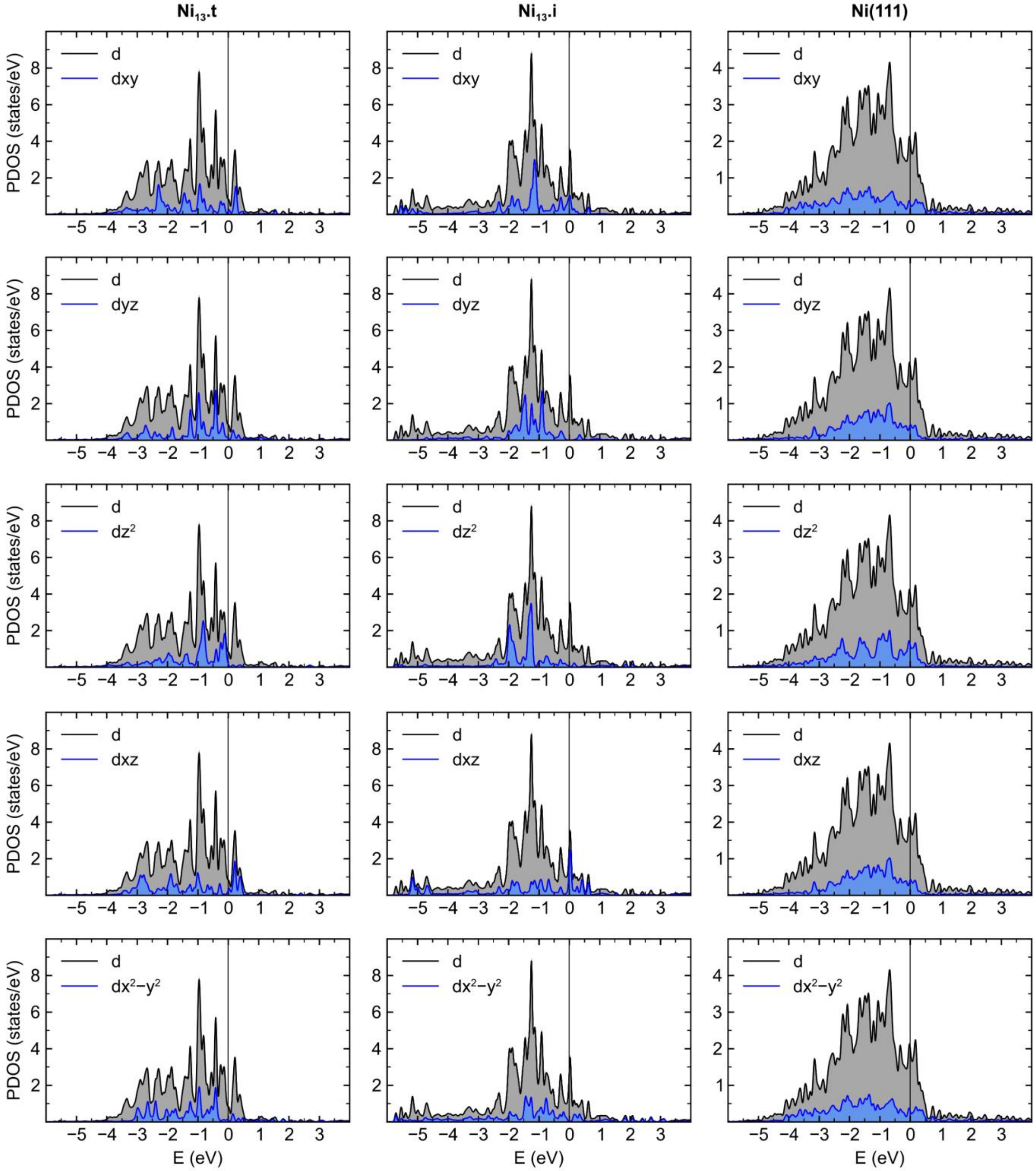

Figure S6. Projected density of states onto the d-states of a Ni atom on the terrace (Ni_{13}.t, left) and at the interface (Ni_{13}.i, middle) of the Ni_{13} cluster, as well as on the Ni(111) surface (right) (cf. Tables S2 and S3). The energy is referred to the Fermi level. The curves are smoothed by a Gaussian level broadening of 0.05 eV.

Table S4. Ni–Ni bond lengths in the first coordination shell of a 9-fold coordinated Ni atom at the Ni(111) surface[1] and a 6-fold coordinated Ni terrace site of the Ni_{13}.CeO_2 system (Ni_{13}.t, cf. Figure S1, Ni atoms forming the Ni_{13}.t–Ni bonds are labelled according to Figure S1), before and after CH_4 adsorption. Changes upon CH_4 adsorption are shown, reflecting the higher flexibility of the ceria-supported Ni_{13} cluster.

Ni-Ni bond	Length (pm)				Change (pm)	
	**Ni(111)**	**Ni_{13}.t**	**CH_4/Ni(111)**	**CH_4/Ni_{13}.t**	**CH_4/Ni(111) − Ni(111)**	**CH_4/Ni_{13}.t − Ni_{13}.t**
**1 ($Ni_{\#8}$)**	249.0	264.9	249.2	308.8	0.2	43.9
**2 ($Ni_{\#6}$)**	249.0	227.6	249.1	232.7	0.1	5.0
**3 ($Ni_{\#11}$)**	249.0	239.2	249.3	241.4	0.2	2.2
**4 ($Ni_{\#10}$)**	249.1	236.0	249.4	234.2	0.3	−1.8
**5 ($Ni_{\#1}$)**	249.1	232.2	249.5	236.1	0.4	4.0
**6 ($Ni_{\#4}$)**	249.0	275.0	249.4	291.8	0.4	16.8
**7**	245.8	-	245.8	-	0.0	-
**8**	245.8	-	245.8	-	0.0	-
**9**	245.9	-	245.9	-	0.0	-
**Average**	**248.0**	**245.8**	**248.2**	**257.5**	**0.2**	**11.7**

Table S4 reveals the higher fluxionality of ceria supported small clusters compared to the extended metal surface. Upon CH_4 adsorption, larger changes in the Ni–Ni bond lengths are observed for the active Ni sites in the Ni_{13} cluster compared to Ni(111) (cf. an average change of 11.7 pm for Ni13.t and 0.2 pm for a surface site of Ni(111)).

CH_4 Dehydrogenation

Table S5. Summary of reaction energies (ΔE) and activation energies (E_a) of all elementary steps involved in CH_4 dehydrogenation (cf. Figures 1, S7 and S8) on $Ni_{13}.CeO_2$ compared with literature values for Ni(111). H atoms resulting from R2 and R3 were removed from the cluster before the calculation of R4.

Number	Equation	ΔE (eV)			E_a (eV)		
		Ni_{13}.i	Ni_{13}.t	Ni(111)	Ni_{13}.i	Ni_{13}.t	Ni(111)
R1	$CH_{4gas} \rightarrow CH_4$	−0.44	−0.36	−0.26,[1] −0.21[2]	0.00	0.00	0.00
R2	$CH_4 \rightarrow CH_3 + H$	0.32	0.02	0.09,[1] 0.31,[2] 0.55[3]	0.34	0.36	0.90,[1] 1.24,[2] 1.21,[3] 1.25,[4] 1.13,[5] 1.32,[6] 1.05,[7] 1.09,[8] 1.18,[9] 0.91,[10] 1.15[11]
R3	$CH_3 \rightarrow CH_2 + H$	0.34	0.18	0.19,[2] 0.33,[3] 0.07[10]	0.68	0.72	0.76[2], 0.87,[3] 0.71,[6] 0.86,[9] 0.70[10]
R4	$CH_2 \rightarrow CH + H$	−0.78	−0.37	−0.21[2], −0.16,[3] −0.34[10]	0.36	0.60	0.38,[2] 0.43,[3] 0.29,[6] 0.46,[9] 0.35,[10]
R5	$CH \rightarrow C + H$	−0.16	−0.63	0.80[2], 0.57,[3] 0.52[10]	0.72	0.53	1.44,[2] 1.45,[3] 1.44,[6] 1.47,[9] 1.33[10]

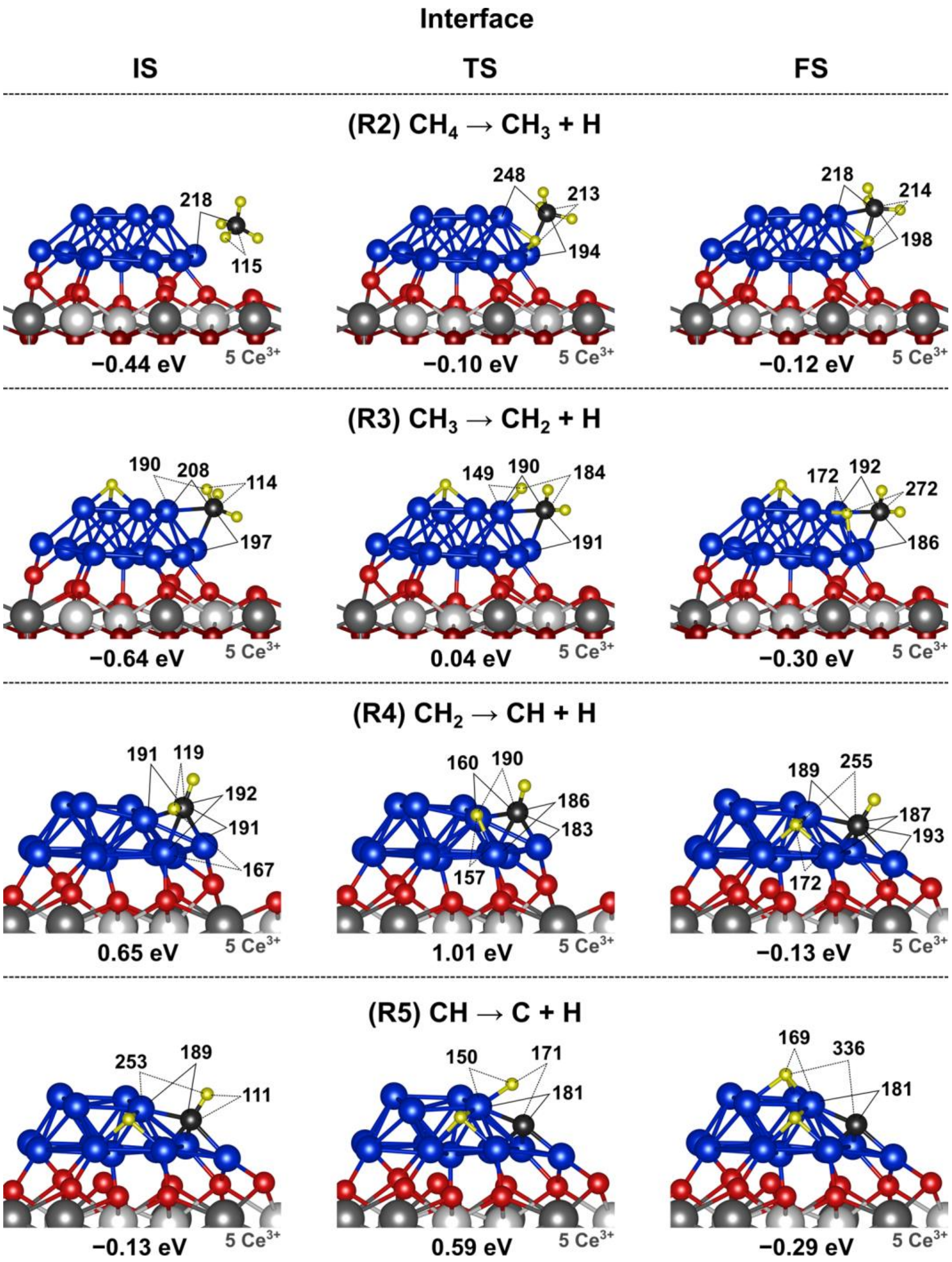

Figure S7. Structures and energies (relative to CH_4 in the gas phase and the clean system) of the initial states (IS), transition states (TS), and final states (FS) for CH_4 dehydrogenation steps on interfacial sites of the Ni_{13} cluster supported on CeO_2(111). Note that two H atoms were removed before R4, which were balanced by adding a free-standing H_2 molecule in the calculation of the energies (E[state] + $E[H_{2gas}]$ – $E[CH_{4gas}]$ – $E[Ni_{13}.CeO_2]$). Selected interatomic distances (in pm) are indicated.

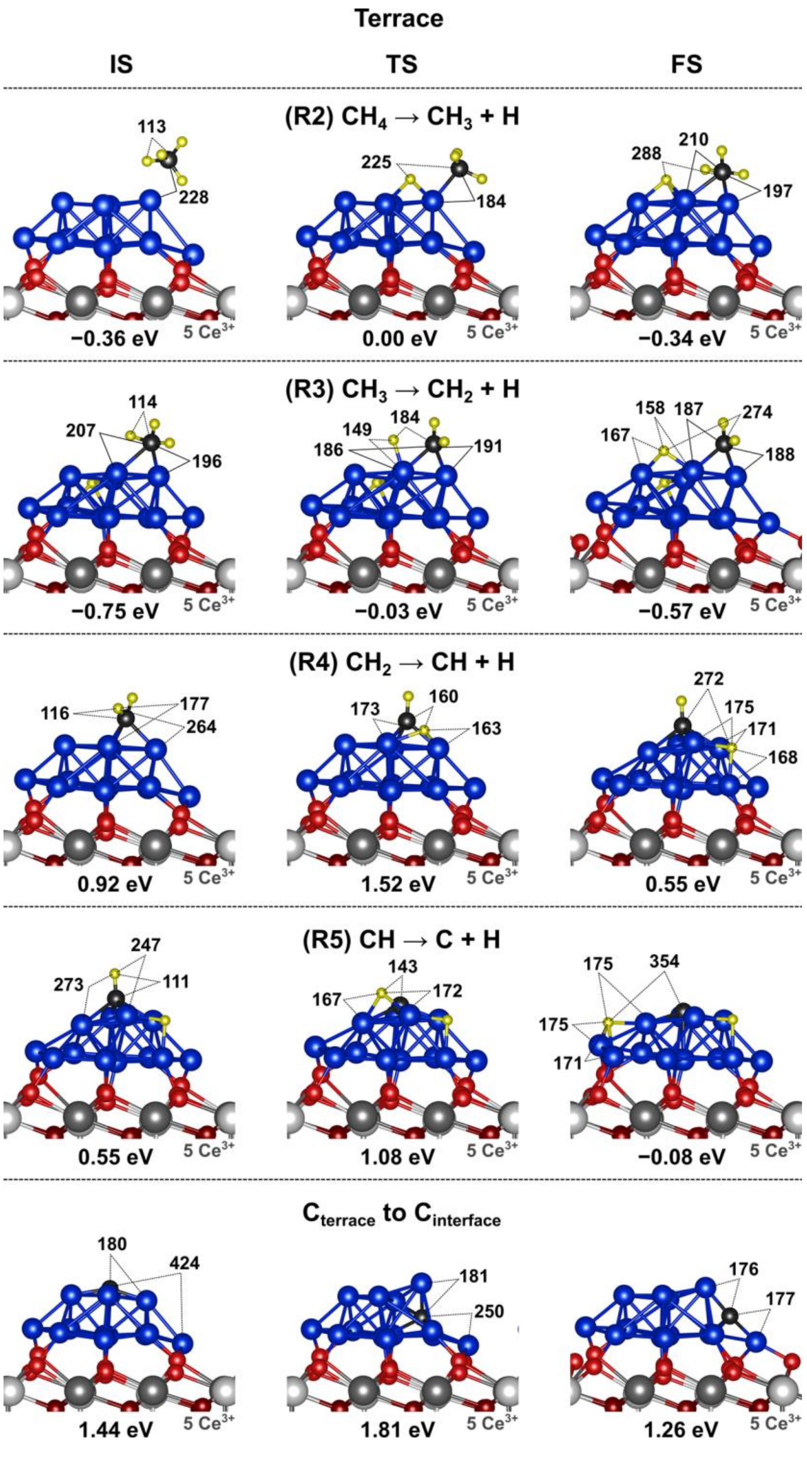

Figure S8. Structures and energies (relative to CH_4 in the gas phase and the clean system) of the initial states (IS), transition states (TS), and final states (FS) for CH_4 dehydrogenation steps on terrace sites of the Ni_{13} cluster supported on CeO_2(111). Carbon migration from terrace to interface sites is also shown (bottom panel). Note that two H atoms were removed before R4 and another two before carbon migration, which were balanced by adding one and two free-standing H_2 molecules in the calculation of the energies, respectively (E[state] + (1 or 2) $E[H_{2gas}]$ – $E[CH_{4gas}]$ – $E[Ni_{13}.CeO_2]$). Selected interatomic distances (in pm) are indicated.

Binding energy of isolated adsorbates

Table S6. Binding energies (*BE*) of isolated chemisorbed CH_x (x=0–4), H, H_2, O, OH, CO and COH species relative to the species in gas phase and the clean systems (for a species A, $BE(A) = E[A/Ni_{13}.CeO_2] - E[A] - E[Ni_{13}.CeO_2]$). For CH_x intermediates, co-adsorbed H atoms were removed in each $CH_4 \rightarrow CH_3 \rightarrow CH_2 \rightarrow CH \rightarrow C$ step (cf. R1–R5 in Table S5, and Figures S7 and S8) and the structures were reoptimized. H species corresponds to the removal of CH_3 from the CH_3 + H states, followed by geometry optimization. CO and COH species correspond to the systems resulting from removing the H atom adsorbed on lattice oxygen (H_s) in the COH intermediate pathway (cf. Figure 4, and R8 and R9 in Figure S10). O and OH species were adsorbed separately on the same sites where they adsorb when formed via the H_2O dehydrogenation pathway (III and II in Figure 7 for O and OH, respectively, i.e., OH corresponds to the 2-fold-bound OH_b species).

Species	Binding energy (eV)		
	**Ni_{13}.i/ CeO_2(111)**	**Ni_{13}.t/ CeO_2(111)**	**Ni(111)**
**CH_4**	−0.44	−0.36	−0.26,[1] −0.21[2]
**CH_3**	−2.57	−2.36	−2.02,[2] −1.81,[9] −1.91,[10] −1.91[12]
**CH_2**	−4.52	−4.25	−4.40,[2] −3.85,[9] −4.01,[10] −3.99[12]
**CH**	−7.20	−6.40	−6.66,[2] −6.35,[9] −6.43[10]
**C**	−7.88	−7.70	−7.03,[2] −6.61,[9] −6.78[10]
**H**	−2.68	−2.77	−2.68,[2] −2.77,[9] −2.81[10]
**H_2**	−0.47	−0.64	−0.38,[2] −0.22[10]
**OH**	−3.97	-	−3.12,[2] −3.11,[9] −3.42[10]
**O**	−5.86	-	−5.94,[2] −5.07,[9] −5.67[10]
**COH**	−5.02	-	−4.95,[2] −4.33,[9] −4.39[10]
**CO**	−2.18	-	−1.98,[2] −1.91,[9] −1.92[10]

OH and O Formation

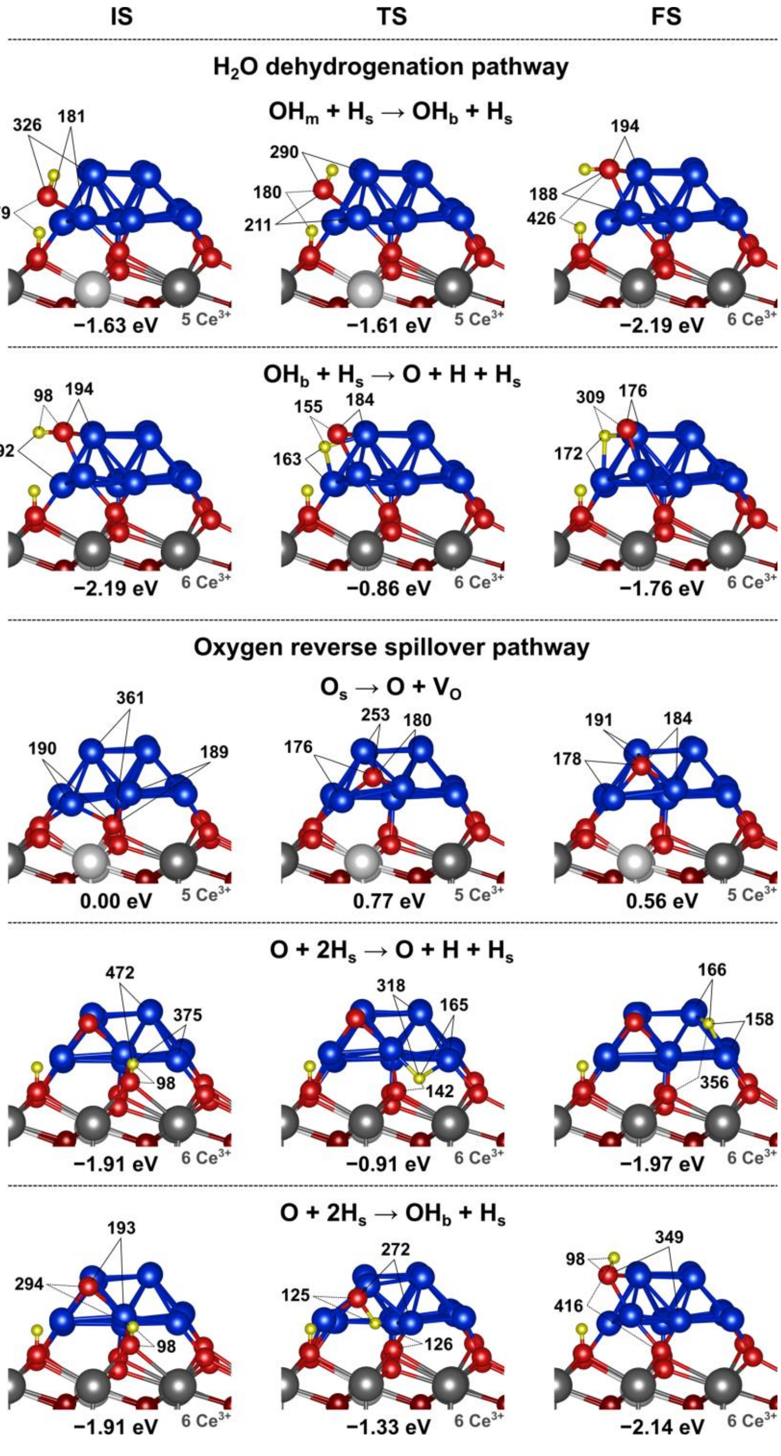

Figure S9. Structures and energies (relative to H_2O in the gas phase and the clean system in all cases but one, namely, the $O_s \rightarrow O+V_O$) of the initial states (IS), transition states (TS), and final states (FS) for steps involved in O and OH formation on the Ni_{13} cluster supported on $CeO_2(111)$. Selected interatomic distances (in pm) are indicated.

CO formation

Table S7. Reaction energies (ΔE) and activation energies (E_a) for CO formation steps evaluated on $Ni_{13}.CeO_2$ (cf. Figure S10) compared with literature values for Ni(111) when available.

Number	Equation	ΔE (eV)		Ea (eV)	
		$Ni_{13}.CeO_2$	Ni(111)	$Ni_{13}.CeO_2$	Ni(111)
R6	$C + O_s \rightarrow CO + Vo$	0.10	-	2.17	-
R7	$C + O \rightarrow CO$	−0.31	−2.50,[2] −1.72,[3] −1.35[10]	1.47	0.59,[2] 1.30,[3] 1.59,[10] 2.07[11]
R8	$C + OH + H_s \rightarrow COH + H_s$	0.43	−0.61,[3] −0.55[10]	0.89	1.14,[3] 1.46[10]
R9	$COH + H_s \rightarrow CO + H + H_s$	−1.01	−0.98,[2] −0.99[10]	0.88	1.14,[2] 0.98[10]

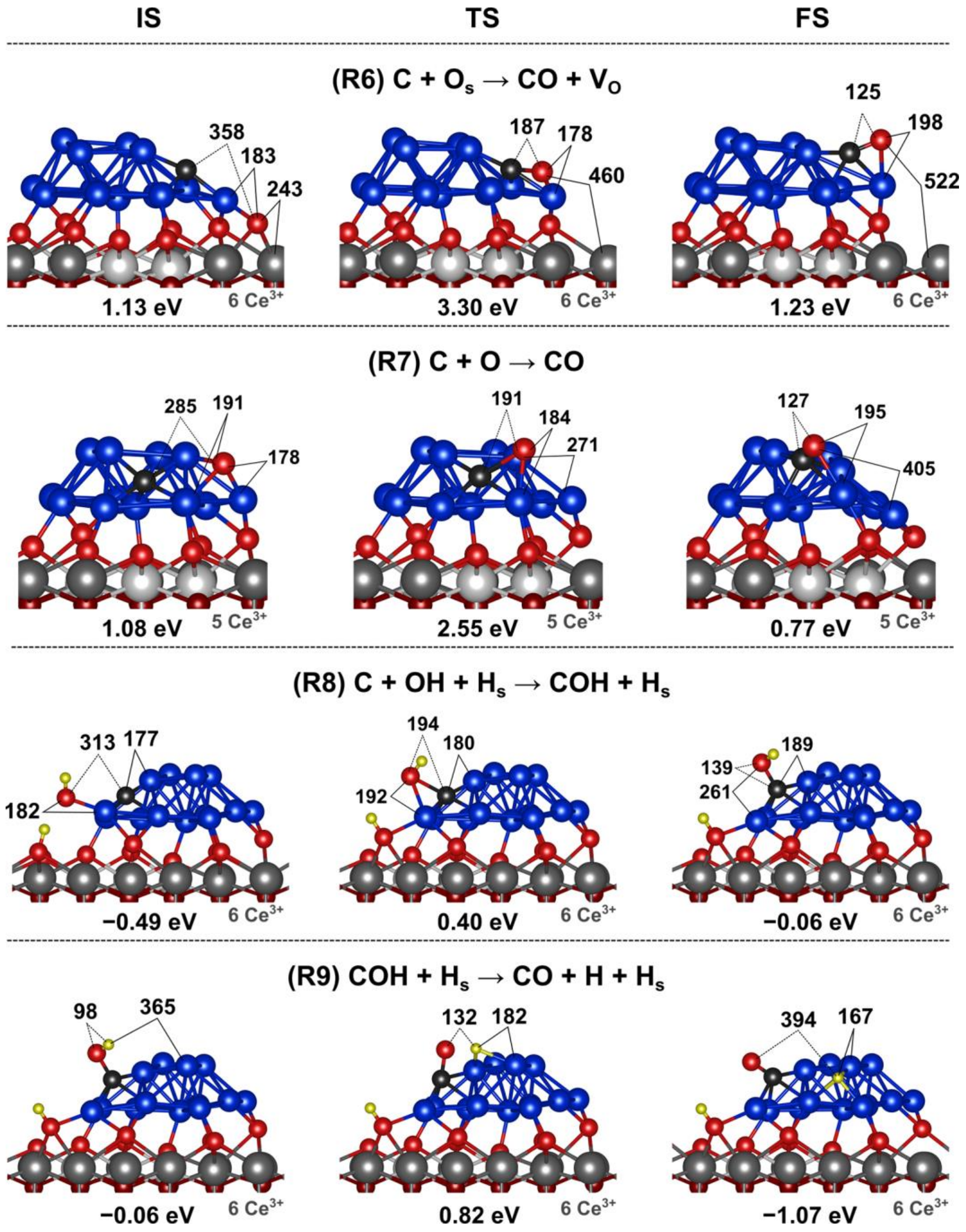

Figure S10. Structures and energies of the initial states (IS), transition states (TS), and final states (FS) for the CO formation steps evaluated on the Ni_{13} cluster supported on $CeO_2(111)$. Selected interatomic distances (in pm) are indicated. Note that in the IS of R6, O_s is lifted and there are 6 Ce^{3+} cations. The energies of IS, TS and FS were calculated according to the stoichiometry of the reaction, i.e., for R6: $E[\text{state}] + 2\,E[H_{2gas}] - E[CH_{4gas}] - E[Ni_{13}.CeO_2]$, for R7: $E[\text{state}] + 3\,E[H_{2gas}] - E[CH_{4gas}] - E[H_2O_{gas}] - E[Ni_{13}.CeO_2]$, and for R8 and R9: $E[\text{state}] + 2\,E[H_{2gas}] - E[CH_{4gas}] - E[H_2O_{gas}] - E[Ni_{13}.CeO_2]$.

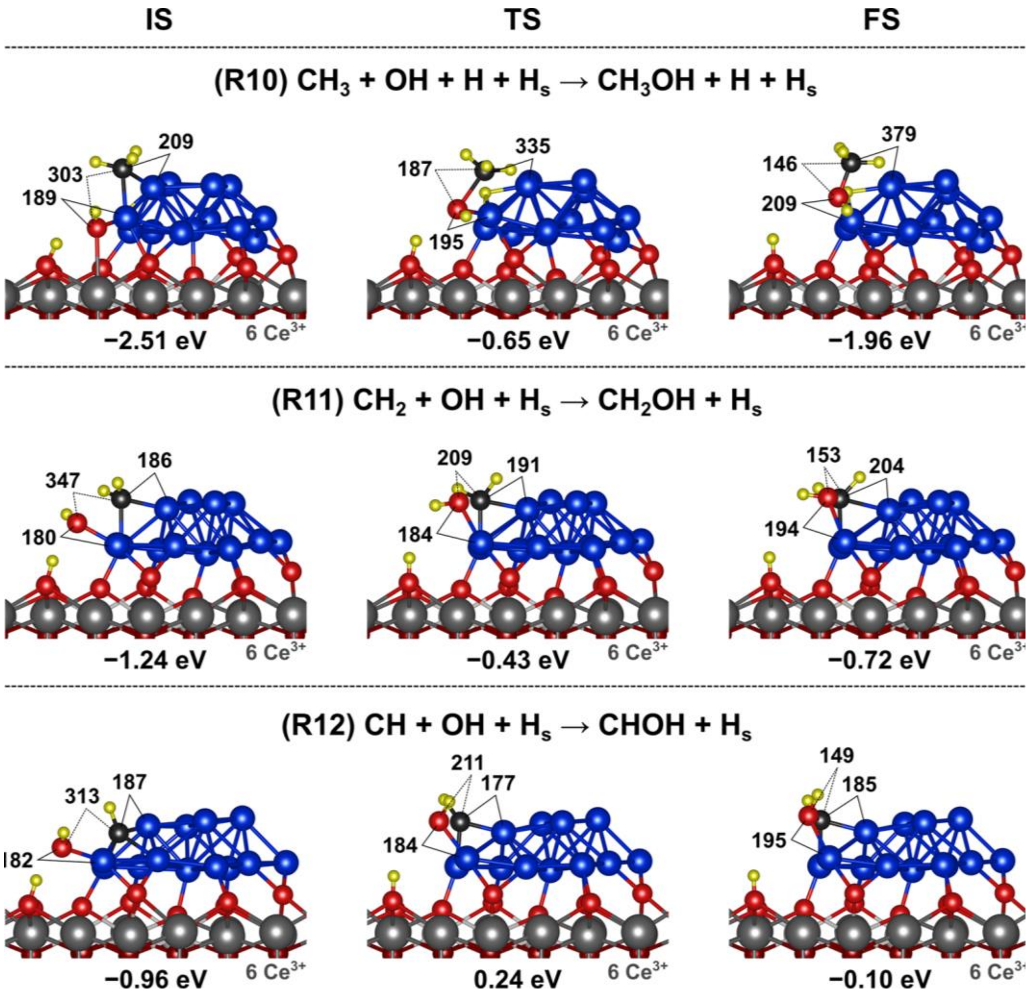

Figure S11. Structures of the initial states (IS), transition states (TS), and final states (FS) for CH_xOH (x=1–3) formation reactions on the Ni_{13} cluster supported on CeO_2(111). Selected interatomic distances (in pm) are indicated. The energies of the IS, TS, and FS states were calculated as E[state] + (3−m/2) $E[H_{2gas}] - E[CH_{4gas}] - E[H_2O_{gas}] - E[Ni_{13}.CeO_2]$ where m is the number of H atoms in the system. Note: a H atom adsorbed on lattice oxygen (H_s) is present but it does not participate in the CH_xOH formation reactions. In R10, the H resulting from the first H abstraction from CH_4 is also present.

H_2 formation

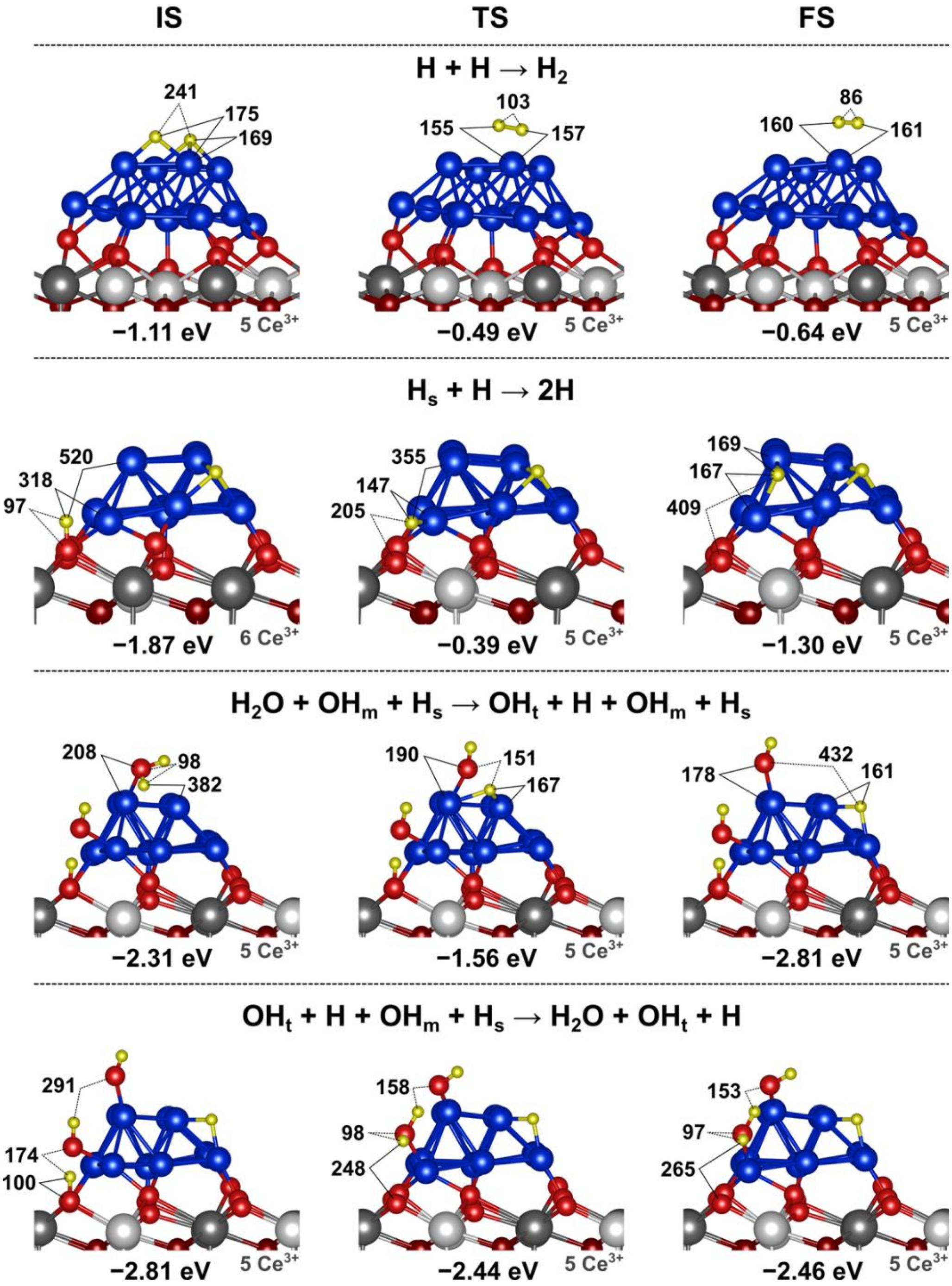

Figure S12. Structures and energies (relative to H_2 in the gas phase in the top two panels and to two H_2O molecules in the gas phase in the bottom two panels, as well as the pristine Ni_{13}.CeO_2 surface) of the initial states (IS), transition states (TS), and final states (FS) for steps involved in H_2 formation on the Ni_{13} cluster supported on CeO_2(111). OH_t indicates binding on the terrace of the Ni_{13} cluster. Selected interatomic distances (in pm) are indicated.

The top panel in Figure S12 shows the formation of H_2 from H species chemisorbed on the Ni_{13} cluster, whereas the second panel shows the direct migration of H_s to the cluster, both described in the main text. The third and fourth panel show an alternative water-assisted pathway. Starting from H_2O dissociative adsorption at the Ni-CeO_2 interface (OH_m + H_s), firstly a second H_2O molecule adsorbs on a Ni^0 terrace site with ΔE_{ads}=−0.68 eV. Subsequently, that molecule dissociates into OH (on the terrace, OH_t) and H, releasing 0.50 eV with a barrier of 0.75 eV (third panel). Finally, H_s migrates towards the OH_m group forming a H_2O molecule at the interface, stabilized by a hydrogen bond with the OH_t group. This last step takes places with ΔE=0.35 eV and an energy barrier of 0.37 eV (fourth panel). After the water molecule is desorbed, the net result is the conversion of OH + H_s into OH + H, that is, the H migration from surface lattice oxygen to the Ni_{13} cluster.

References

(1) Lustemberg, P. G.; Zhang, F.; Gutiérrez, R. A.; Ramírez, P. J.; Senanayake, S. D.; Rodriguez, J. A.; Ganduglia-Pirovano, M. V. Breaking Simple Scaling Relations through Metal–Oxide Interactions: Understanding Room-Temperature Activation of Methane on M/CeO_2 (M = Pt, Ni, or Co) Interfaces. *J. Phys. Chem. Lett.* **2020**, *2* (111), 9131–9137. https://doi.org/10.1021/acs.jpclett.0c02109.

(2) Han, Z.; Yang, Z.; Han, M. Comprehensive Investigation of Methane Conversion over Ni(111) Surface under a Consistent DFT Framework: Implications for Anti-Coking of SOFC Anodes. *Appl. Surf. Sci.* **2019**, *480*, 243–255. https://doi.org/10.1016/j.apsusc.2019.02.084.

(3) Niu, J.; Wang, Y.; Qi, Y.; Dam, A. H.; Wang, H.; Zhu, Y.-A.; Holmen, A.; Ran, J.; Chen, D. New Mechanism Insights into Methane Steam Reforming on Pt/Ni from DFT and Experimental Kinetic Study. *Fuel* **2020**, *266*, 117143. https://doi.org/10.1016/j.fuel.2020.117143.

(4) Burghgraef, H.; Jansen, A. P. J.; van Santen, R. A. Methane Activation and Dehydrogenation on Nickel and Cobalt: A Computational Study. *Surf. Sci.* **1995**, *324* (2–3), 345–356. https://doi.org/10.1016/0039-6028(94)00716-0.

(5) Kratzer, P.; Hammer, B.; No/rskov, J. K. A Theoretical Study of CH_4 Dissociation on Pure and Gold-alloyed Ni(111) Surfaces. *J. Chem. Phys.* **1996**, *105* (13), 5595–5604. https://doi.org/10.1063/1.472399.

(6) Watwe, R. M.; Bengaard, H. S.; Rostrup-Nielsen, J. R.; Dumesic, J. A.; Nørskov, J. K. Theoretical Studies of Stability and Reactivity of CH_x Species on Ni(111). *J. Catal.* **2000**, *189* (1), 16–30. https://doi.org/10.1006/jcat.1999.2699.

(7) Bengaard, H. S.; Nørskov, J. K.; Sehested, J.; Clausen, B. S.; Nielsen, L. P.; Molenbroek, A. M.; Rostrup-Nielsen, J. R. Steam Reforming and Graphite Formation on Ni Catalysts. *J. Catal.* **2002**, *209* (2), 365–384. https://doi.org/10.1006/jcat.2002.3579.

(8) Abild-Pedersen, F.; Greeley, J.; Nørskov, J. K. Understanding the Effect of Steps, Strain, Poisons, and Alloying: Methane Activation on Ni Surfaces. *Catal. Lett.* **2005**, *105* (1–2), 9–13. https://doi.org/10.1007/s10562-005-7998-9.

(9) Wang, S.-G.; Cao, D.-B.; Li, Y.-W.; Wang, J.; Jiao, H. CO_2 Reforming of CH_4 on Ni(111): A Density Functional Theory Calculation. *J. Phys. Chem. B* **2006**, *110* (20), 9976–9983. https://doi.org/10.1021/jp060992g.

(10) Zhu, Y.-A.; Chen, D.; Zhou, X.-G.; Yuan, W.-K. DFT Studies of Dry Reforming of Methane on Ni Catalyst. *Catal. Today* **2009**, *148* (3–4), 260–267. https://doi.org/10.1016/j.cattod.2009.08.022.

(11) Blaylock, D. W.; Ogura, T.; Green, W. H.; Beran, G. J. O. Computational Investigation of Thermochemistry and Kinetics of Steam Methane Reforming on Ni(111) under Realistic Conditions. *J. Phys. Chem. C* **2009**, *113* (12), 4898–4908. https://doi.org/10.1021/jp806527q.

(12) Fajín, J. L. C.; Gomes, J. R. B.; D. S. Cordeiro, M. N. Mechanistic Study of Carbon Monoxide Methanation over Pure and Rhodium- or Ruthenium-Doped Nickel Catalysts. *J. Phys. Chem. C* **2015**, *119* (29), 16537–16551. https://doi.org/10.1021/acs.jpcc.5b01837.

(13) Wang, S.-G.; Liao, X.-Y.; Hu, J.; Cao, D.-B.; Li, Y.-W.; Wang, J.; Jiao, H. Kinetic Aspect of CO_2 Reforming of CH_4 on Ni(111): A Density Functional Theory Calculation. *Surf. Sci.* **2007**, *601* (5), 1271–1284. https://doi.org/10.1016/j.susc.2006.12.059.
